# Mechanistic Investigation of Visible-Light-Driven Photocatalysis in Bio-Synthesized Graphitic Carbon Nitride/MgO Nanocomposites Toward Safranine Orange Dye

**DOI:** 10.1021/acsomega.6c03815

**Published:** 2026-08-13

**Authors:** Priyanka Panchal, Protima Rauwel, Laura Coconubo-Guio, Glemarie Hermosa, Shubham Gautam, Satya Pal Nehra, Elias Estephan, Erwan Rauwel

**Affiliations:** † Institute of Veterinary Medicine and Animal Sciences, 85334 Estonian University of Life Sciences, Tartu 51006, Estonia; ‡ Department of Aeronautical Engineering, 630571 Estonian Aviation Academy, Tartu 61707, Estonia; § Materials Research Center, 232469 Malaviya National Institute of Technology, Jaipur 302017, India; ∥ Center of Excellence for Energy and Environmental Studies, Deenbandhu Chhotu Ram University of Science and Technology, Sonipat 131039, India; ⊥ Laboratoire Bioingénierie et Nanoscience (LBN), 233864 University of Montpellier, Montpellier 34090, France; # Faculty of Engineering, Sagesse University, P.O. Box 50501 Furn El Chebbak, Baabda, Lebanon

## Abstract

In this work, we report a sustainable strategy for the synthesis of magnesium oxide (MgO) nanoparticles, graphitic carbon nitride (g-C_3_N_4_), and MgO/g-C_3_N_4_ nanocomposites derived from orange peel waste, with MgO loadings ranging from 28 to 76 wt %. Comprehensive physicochemical characterization, including optical spectroscopy, structural analysis, and electron microscopy, confirmed the successful formation of well-dispersed heterostructures with tailored surface functionalities. The photocatalytic performance of the as-prepared materials was evaluated toward the degradation of Safranin O under simulated solar irradiation. Among all samples, the nanocomposite containing 61 wt % MgO exhibited the highest activity, achieving 89% degradation within 120 min. Kinetic investigations revealed a rate constant approximately 4.6 and 3.7 times higher than those of pristine g-C_3_N_4_ and MgO, respectively, highlighting the strong synergistic interaction between both components. Importantly, cytotoxicity assays conducted on human dental pulp stem cells demonstrated the good biocompatibility of the synthesized materials. Overall, this study presents a green and scalable route to engineer efficient and biocompatible photocatalysts from biowaste, offering promising prospects for sustainable water-remediation applications.

## Graphical abstract

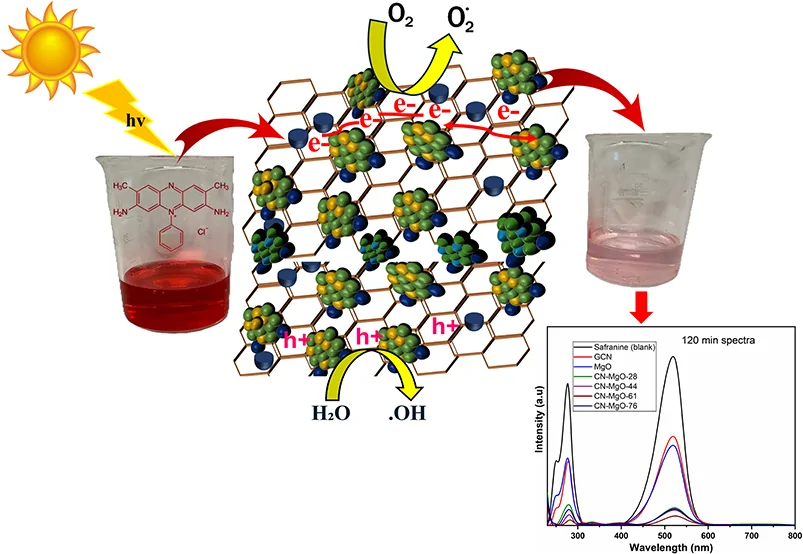

## Introduction

Nanostructured materials such as zeolites, activated carbon, carbon nanotubes, polymer-based nanomaterials, and graphene-derived materials have been extensively studied for water remediation.^1–4^ In particular, graphitic carbon nitride (GCN) has been widely investigated as an efficient material for the same, via photocatalytic degradation processes. As a metal-free, polymeric n-type semiconductor, GCN exhibits a band gap of approximately 2.7 eV, remarkable chemical stability under ambient conditions, no known toxicity, and strong visible light absorption capability, making it a cost-effective material for environmental applications.^5–9^ GCN is generally synthesized through the thermal polymerization of nitrogen-rich precursors such as urea, melamine, dicyandiamide, and thiourea.^9–11^ These precursors undergo condensation at elevated temperatures (450–600 °C), leading to the formation of a layered graphitic structure composed of tri-s-triazine units.^2–4,10^ The synthesis route plays a crucial role in controlling the surface area, porosity, crystallinity, and photocatalytic performance of the resulting material. For example, melamine calcined at 500–550 °C yields more crystalline bulk GCN, whereas urea treated at 500–600 °C produces more porous and defect-rich GCN due to rapid NH_3_ and CO_2_ evolution during polymerization.^12–14^ Pure GCN has demonstrated photocatalytic activity against various organic dyes under UV–vis or visible irradiation. For example, thermally polymerized GCN obtained by calcination of nitrogen-rich precursors (e.g., melamine or urea) at 450–600 °C for 2–4 h has achieved degradation efficiencies of 50–80% for methylene blue, rhodamine B, and methyl orange dyes under visible light within intervals of 60–180 min.^15–18^ Recently, green and sustainable synthesis approaches have also been explored, including the use of plant-part extracts to prepare nanoparticles and their hybrids. Plant phytochemicals serve as natural reducing and stabilizing agents, facilitating nanoparticle formation without the use of hazardous chemicals, while biobased synthesis routes are environmentally benign, renewable, and potentially cost-effective, aligning with sustainable development goals.^19–25^ Therefore, when GCN is synthesized using naturally available plant resources, and since GCN is composed of organic elements that are readily found in plant biomass, it eliminates the use of chemical reagents like urea and melamine, minimizing environmental impacts and thereupon leading to an enhanced sustainable process.^26,27^

Nevertheless, the photocatalytic performance of GCN is limited due to several factors, including rapid recombination of photogenerated electron–hole pairs, moderate visible-light absorption, and relatively low surface area owing to agglomeration. These intrinsic limitations therefore require further enhancement through suitable modification strategies.^15–18,28–31^ In this context, coupling GCN with semiconductor-based metal/metal oxide (e.g., Ag, CuO, TiO_2_, ZnO and MgO) to promote heterojunction formation in order to overcome the problem of rapid recombination of photogenerated charge carriers and in turn, improves the photocatalytic activity. This strategy allows degrading a wide range of dyes-including methylene blue, rhodamine B, methyl orange, and Congo red-under visible light, with a high efficiency of 85–99% removal in a time span of 30–120 min.^15–18,32–39^ In that regard, MgO nanoparticles have spurred interest due to their possible application as pollutant absorbents, photocatalysts, and antimicrobial agents. Although MgO possesses a very wide band gap (∼4–7 eV), which limits its intrinsic absorption mostly to the UV region, it can still function as a photocatalyst when reduced to a nanoparticle and absorb visible light due to the availability of a high number of active sites on its surface. These active sites correspond to oxygen vacancies or F centers that tend to create bandgap or defect states.^40–44^ Pure MgO nanoparticles have demonstrated ∼40–90% degradation efficiency of dyes such as methylene blue, methyl orange, crystal violet, brilliant blue, and brilliant green under visible irradiation, indicating promising photocatalytic performance.^10,35–40^ Moreover, MgO doped GCN nanocomposites demonstrate high photocatalytic efficiency of up to 80–98% against methylene blue, methyl orange, crystal violet, eosin yellow, rhodamine B, under visible and simulated sunlight. Most of these studies however focus on chemically synthesized materials and their photocatalytic activity. Therefore, systematic investigations on green synthesized nanocomposites, including biocompatibility, as well as MgO loading optimization are limited. Addressing these gaps could in principle, improve both photocatalytic performance and potential environmental applications.^10,33,41–50^

In this study, MgO and GCN nanoparticles, and GCN-MgO nanocomposites were synthesized using orange peel biowaste as a sustainable reducing and stabilizing agent. The synthesized nanomaterials were characterized using XRD for crystal structure and phase analysis. Raman spectroscopy, XPS, and FTIR were applied to study their chemical bonding, while SEM and TEM provided their morphology and particle size. UV–vis and photoluminescence (PL) spectroscopy helped elucidate their optical properties. The cytotoxicity of the synthesized nanomaterials was also investigated, and revealed a cell viability of up to 90%, indicating an excellent biocompatibility, advocating their safety for environmental applications. The photocatalytic study showed an enhanced activity, achieving up to 89% degradation against Safranine O (SO) dye under visible light solar-simulator exposure.

A comparison between chemically synthesized and biological orange-fruit peel-assisted GCN synthesis highlights notable differences in structure, composition, and photocatalytic behavior. Chemically synthesized GCN, typically prepared via conventional thermal or polymerization of pure precursors, generally exhibits a more ordered polymeric structure with higher crystallinity and more complete condensation of heptazine units, resulting in a relatively well-defined electronic structure and controlled charge transport properties. Such structures have been widely reported to show efficient photocatalytic activity for hydrogen evolution and pollutant degradation, as demonstrated by He et al., and Yang et al., Wang et al.^2,3,5^ In contrast, biologically derived GCN obtained via orange-fruit peel-assisted routes is characterized by higher defect density, partial condensation of heptazine units, and less ordered stacking due to naturally occurring organic constituents in biomass precursors. These structural features introduce defect states that can modify the electronic structure, and facilitate charge trapping and migration. As a result, although green-synthesized GCN may possess lower crystallinity, it can exhibit comparable or even enhanced photocatalytic performance in certain cases due to defect-assisted active sites. Moreover, the green synthesis route also offers significant advantages in terms of sustainability, cost-effectiveness, and waste valorization compared to conventional chemical methods.

## Materials and Methods

### Materials

The chemicals employed are all of analytical grades. Thermo Fischer chemicals include magnesium acetate precursor (99% purity), Safranine O dye (95% purity), ethylene diamine tetra acetic acid (EDTA-98% purity), silver nitrate (99.8% purity), isopropyl alcohol (99% purity), and orange-peel collected from household waste. Human dental pulp stem cells (HDPSC) for cytotoxicity were obtained from the permanent teeth of patients from Health Sciences Faculty of Odontology at the University of Montpellier (France) with the agreement of the Ethical Committee of the Health Sciences Faculty of Odontology at the University of Montpellier, France.

### Characterization

X-ray diffraction (XRD) studies were conducted using a Bruker D8 Advance diffractometer equipped with CuK_α1_ radiation (λ = 0.15406 nm), in the range of 2θ angle from 20̊-80̊ to determine the crystalline structure selected by a Ge (111) monochromator. Morphology of NPs and NCs were performed using a scanning electron microscope (SEM) Zeiss Merlin (Carl Zeiss Microscopy, Munich, Germany). The microstructure of the samples was characterized using of transmission electron microscope HR-TEM (Tecnai 20 G2 200 kV, S-TWIN). Raman spectra were collected using a WITec Confocal Raman Microscope System alpha 300R (WITec Inc., Ulm, Germany). Excitation in confocal Raman microscopy was generated using a frequency-doubled Nd:YAG laser (Newport, Irvine, CA, USA) at wavelengths of 532 nm with 50 mW maximum laser output power in a single longitudinal mode. Each Raman spectrum was acquired with an integration time of 10 s per scan and 10 accumulations. Fourier transform infrared (FTIR) Spectrometer (Nicolet is10 Thermo Scientific, Driesch, Germany) was used to study the chemical bonds in the samples in the range of 400 to 4000 cm^–1^. XPS measurements were performed at room temperature with (ESCA + Omicron Nanotechnology Oxford Instrument Germany) in a base pressure of 5 × 10^–10^ mbar using monochromatic Al K-alpha radiation (1486.7 eV) as an excitation source operated at 300 W. The energy resolution as measured by the fwhm of the Ag 3d5/2 peak for a sputtered silver foil was 0.60 eV. The spectra were calibrated relative to the C 1s at 284.8 eV. The optical absorbance of the biosynthesized MgO, GCN, and CN-MgO nanocomposites were recorded with UV–vis spectrophotometer (Ocean Optics, USA) in the range of 200 to 1100 nm. Photoluminescence (PL) study was carried out on MgO powders at room temperature with an excitation wavelength of 365 nm of LSM-365A LED (Ocean insight, Orlando, FL, USA) with a specified output power of 10 mW.

### Materials Synthesis

#### Plant Extract

Thirty g of orange peels (OP) were cleaned first with tap water to remove dirt and soil, then with distilled water (DW) to further decontaminate. Then OP was chopped into smaller pieces and placed in a 200 mL borosilicate beaker. Then 50 mL double-distilled water (DDW) was added to the beaker containing the chopped OP. The mixture of chopped OP and double-distilled water was boiled until the solution turned dark orange. After boiling, the supernatant was separated from the solid residue by pouring it through filter paper as shown in Figure 1a. The resulting filtrate, was then stored at 4 °C for subsequent use.

**Figure 1:**
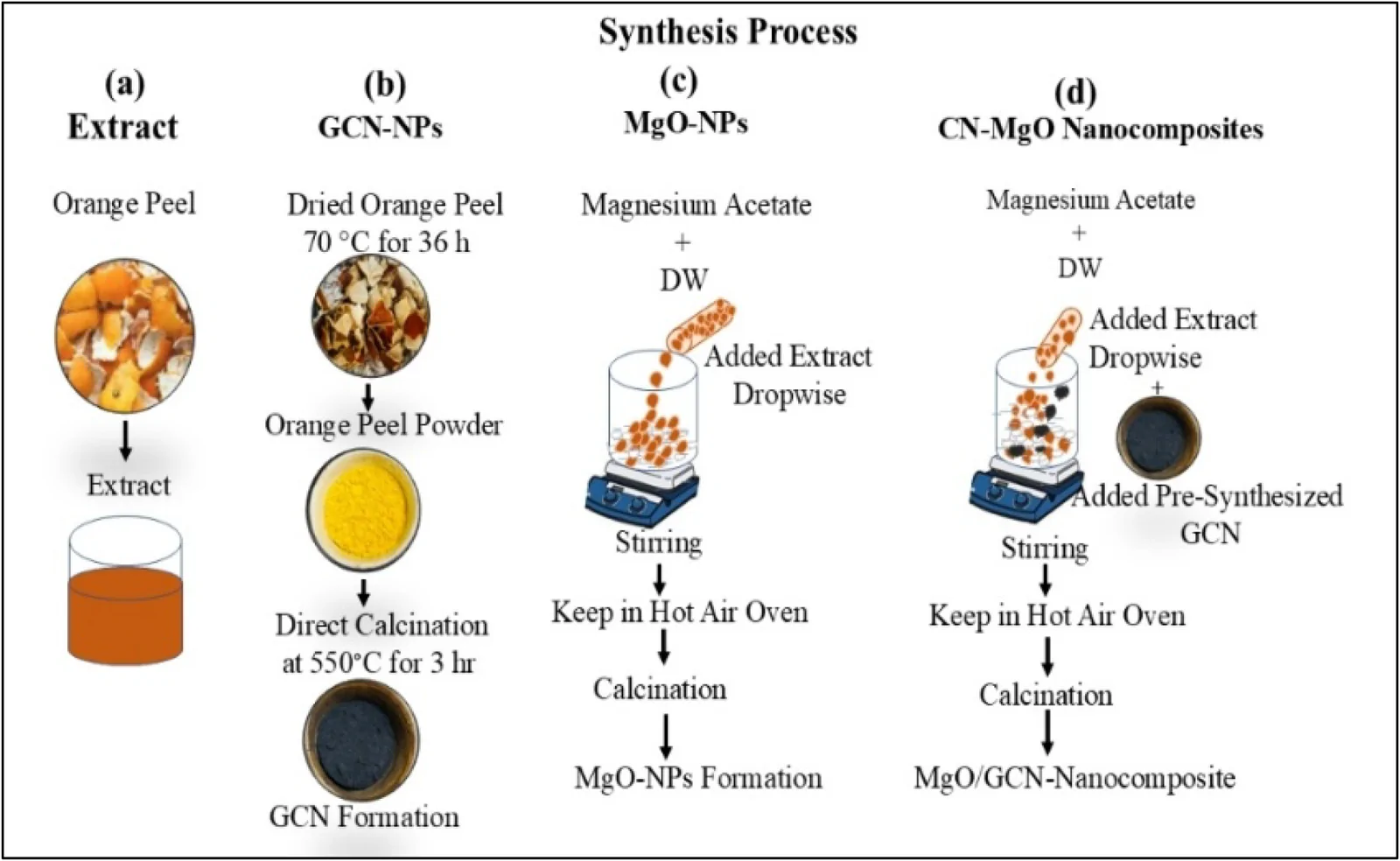
Synthesis process (a) preparation of orange peel extract, (b) synthesis of GCN, (c) synthesis of MgO, and (d) synthesis of CN-MgO nanocomposites.

#### Orange Peel (OP) Biowaste-Derived Nitrogen-Rich Graphitic Carbon (GCN)

Orange-fruit peel (OP) biowaste derived nitrogen rich graphitic carbon (GCN): The graphitic carbon nitride (GCN) was synthesized from orange-fruit peel (OP) through a systematic thermal polymerization process. Initially, fresh orange peels were thoroughly washed, dried in a hot air oven at 70 °C for 36 h, and subsequently ground into a fine powder. ∼30 g of the dried OP powder was then transferred into a covered ceramic crucible to maintain a controlled thermal environment during carbonization and polymerization. The crucible was placed in a muffle furnace and heated to 550 °C at a controlled heating rate of 2 °C min^–1^, followed by calcination for 3 h under atmospheric conditions. During the thermal treatment, the organic constituents of orange peel underwent progressive dehydration, condensation, and thermal polymerization, leading to the formation of intermediate carbonaceous nitrogen-rich structures and subsequent development of the layered graphitic GCN framework. After cooling to room temperature, the obtained GCN product was gently crushed using a mortar and pestle to obtain a homogeneous fine powder. A detailed schematic representation illustrating the precursor transformation, thermal polymerization conditions, intermediate structural evolution, and final formation of GCN has been included in the revised manuscript Figure 1b to improve methodological clarity and reproducibility.

#### Orange Peel Biowaste-Mediated Magnesium Oxide Nanoparticles (MgO-NPs)

Magnesium acetate tetrahydrate was dissolved in 50 mL of double-distilled water (DDW). The solution was stirred at room temperature for 1 h. After that, 15 mL of the OP extract was added drop by drop to the solution. The appearance of a slight yellow color after the addition of the OP extract suggests the initiation of MgO-NP precipitation. The plant extract likely contains bioactive compounds that act as reducing and stabilizing agents for the NPs synthesis. The solution was left for stirring for an additional 2 h. After synthesis, the solution was centrifuged at 5000 rpm for 15 min. The pellet composed of MgO-NPs was collected after centrifugation. It was then dried in a hot air oven at 60 °C for 12 h as shown in Figure 1c. The dried sample was then transferred to a crucible and it was kept for calcination for about 3 h at 350 °C.

#### Orange Peel Biowaste-Mediated Magnesium-Loaded Graphitic Carbon Nitride Nanocomposites

CN-MgO nanocomposites were synthesized as follow: aqueous solution of magnesium acetate tetrahydrate with concentrations of 0.05, 0.1, 0.2, and 0.4 M, were prepared by dissolving the precursor in 100 mL of distilled water in four different containers labeled with a, b, c and d followed by the continuous addition of 10 mL OP extract drop by drop and under continuous stirring for 2 h at room temperature. After stirring process 0.5 g of presynthesized CN-MgO were added in the above solution in the 4 containers, and again stirred for 1 h. The centrifugation of all solutions at 6000 rpm for 5 min was carried out to separate the solid particles that were further washed with isopropyl alcohol to remove the last impurities. The obtained samples were kept in a hot-air oven for drying at 80 °C for 8 h. The CN-MgO obtained exhibited various colors, i.e., black (CN-MgO-28), dark gray (CN-MgO-44), gray (CN-MgO-61), and light gray (CN-MgO-76). All four samples were then heated at 350 °C for 3 h. The reported MgO contents (28%, 44%, 61%, and 76%) represent nominal compositions estimated from the relative precursor concentrations used during synthesis. The composition of the produced CN-MgO correspond to MgO contents of 28%, 44%, 61%, and 76%, which were obtained using 0.05, 0.1, 0.2, and 0.4 M precursor solutions, respectively as shown in Figure 1d and listed in Table 1.

**Table 1:** List of Synthesized Nanoparticles and Their Nanocomposites

Name of material	GCN (g)	Magnesium acetate precursor (M)	MgO quantity (%)
**GCN**	----	00	00
**MgO**	0	1	100
CN-MgO-28	0.5	0.05	28
CN-MgO-44	0.5	0.1	44
CN-MgO-61	0.5	0.2	61
CN-MgO-76	0.5	0.4	76

### Cytotoxicity Assay

The cytotoxicity of the biosynthesized NPs and nanocomposites was assessed by using the Trypan blue dye exclusion method on human dental pulp stem cells (HDPSC) procured from the permanent teeth of patients. HDPSC were cultivated under conventional cell culture conditions (37 °C, 5% CO_2_) in 24-well plates (Falcon), using full culture media comprising α-MEM (Gibco) supplemented with 10% fetal bovine serum (Sigma) and 1% penicillin–streptomycin (Sigma) until achieving 80% confluence. Subsequently, cells were subjected to 50 μg/mL concentrations of the biosynthesized compounds. Cell viability is assessed using trypan blue (Invitrogen) to evaluate cytotoxic effects. In summary, for each condition, cells were detached using Trypsin-EDTA (Gibco), which was then inactivated by full culture media. From each cell suspension, 10 μL were combined with an equal proportion of trypan blue and placed onto Countess chamber slides (Invitrogen). Cell enumeration was conducted by the automated cell counter Countess 3 (Thermo Fisher).

### Dye Degradation

Dye degradation of SO was performed under a solar simulator (1 Sun irradiance). For each experiment, 5 mg of photocatalyst was used in 100 mL of solution containing 5 ppm of SO that was stirred uniformly using a magnetic stirrer for 30 min in dark condition to promote the adsorption and only study the degradation properties. Then the first reading in dark, followed by six readings were performed at intervals of 20 min of light exposure to control the photocatalytic degradation of the dye. All measured samples were previously centrifuged at 2000 rpm for 3 min before measuring the absorption with a UV–vis spectrophotometer in the range of 200 to 800 nm in order to estimate the remaining dye concentration in the sample.

The photocatalytic degradation kinetics of the dye were analyzed using the Langmuir–Hinshelwood kinetic model, which is commonly applied to heterogeneous photocatalytic systems. In this mechanism, the degradation reaction occurs on the catalyst surface and essentially involves two steps: the adsorption of dye molecules onto the photocatalyst surface and their oxidation by photogenerated reactive species. The synthesized nanocomposites have abundant active sites that promote adsorption of dye molecules, which is consistent with the 30 min adsorption step performed in dark, prior to irradiation. Therefore, the Langmuir- Hinshelwood model is suitable for describing the photocatalytic behavior of the nanoparticles and nanocomposites. The Langmuir–Hinshelwood equation simplifies to a pseudo-first-order kinetic expression.^15,17,51,52^

i. Degradation percentage = 1-[C/C_0_] *100
ii. Pseudo first order reaction kinetics = In C_0_/C = Kt

Where, C is Concentration of dye at t, C_0_ is the initial concentration of the dye at t = 0, and K is the rate constant in min^–1^.

### Scavenger Experiments

Scavenger experiments were conducted to identify the active Reactive Oxygen Species responsible for the photocatalytic degradation of SO dye. The experiments were performed under the same conditions used for the photocatalytic degradation studies. After achieving equilibrium, specific scavengers were added individually to the reaction mixture to quench different reactive species. Ethylene diamine tetra acetic Acid (EDTA, 10 mM) was used as a scavenger for photogenerated holes (h^+^), Isopropyl Alcohol (IPA, 10 mM) was used as a hydroxyl radical (•OH) scavenger, and Silver Nitrate (AgNO_3_, 1 mM) was used as an electron (e^–^) scavenger.^15,16,45^

## Results and Discussion

### Crystallographic Analysis with XRD

The XRD patterns of the biosynthesized MgO nanoparticles, GCN, and CN-MgO nanocomposites revealed distinct diffraction peaks matching standard data as shown in Figure 2a–f. Here, the XRD pattern of MgO nanoparticles shows, Figure 2a, peaks at 36.70°, 42.41°, 61.67°, and 77.79° corresponding to the (111), (200), (220), and (222) planes of the cubic-phase of MgO. The intensity of these peaks and the absence of other peaks highlight the high crystallinity and purity of the MgO-NPs (JCPDS 97-7746).^22,23,40–44^ To estimate the crystallite sizes (*D*) of MgO-NPs, the Debye–Scherrer formula fwhm (full-width at half maxima) of the intense diffraction peak corresponding to the (200) reflection was used, *D* = 0.9λ/βcosθ as shown in Table 2.^15,23,43,45,49^ The identification of CN-MgO pattern is known to be challenging as XRD peak positions can vary from one phase to another.^15,16,18,27,39,46^ In case of pure GCN, the XRD patterns in Figure 2b, exhibit a strong XRD peak at 2θ of 27.25° that corresponds to the (002) plane, common to all GCN phases with the interlayer spacing of d = 3.24 Å (JCPDS file No. 87–1526). Other peak in pure GCN around 24̊ at 2θ angle corresponds to carbon/graphite mixtures.^49,50^ The presence of the characteristic (002) diffraction peak together with the existing FTIR, Raman, and XPS results (see here after) supports the formation of a graphitic carbon nitride framework rather than simple carbonized biomass. The XRD patterns of the nanocomposites, Figure 2c–f only XRD peaks at 42.86°, 62.18°, and 78.20° are visible and correspond to (200), (220), and (222) planes of the MgO cubic structure. The shift of peaks toward higher 2θ values indicates a decrease in lattice spacing due to compressive strain in the MgO lattice caused by strong interfacial interaction with GCN.^53^ This strain arises from lattice mismatch between the two phases, leading to lattice contraction and peak displacement. It also partially overlaps with the GCN peak, which shifts slightly from 27.25° to 27.23°. At the highest incorporation of MgO in CN-MgO-76, the intensity decrease is more pronounced, though the GCN peak remains visible in all nanocomposite, consistent with the smallest nanoparticle size of 13 nm. These results confirm the formation of CN-MgO nanocomposites, in which MgO nanoparticles enhance the structural features without altering the fundamental crystalline nature of the original materials.

**Table 2:** Crystallite Size of MgO, GCN Nanoparticles, and CN-MgO Nanocomposites Using Debye-Scherrer Formula

Name of material	Crystallite size (nm)
**GCN**	—
**MgO**	20.98
CN-MgO-28	18.94
CN-MgO-44	16.36
CN-MgO-61	19.03
CN-MgO-76	13.14

**Figure 2:**
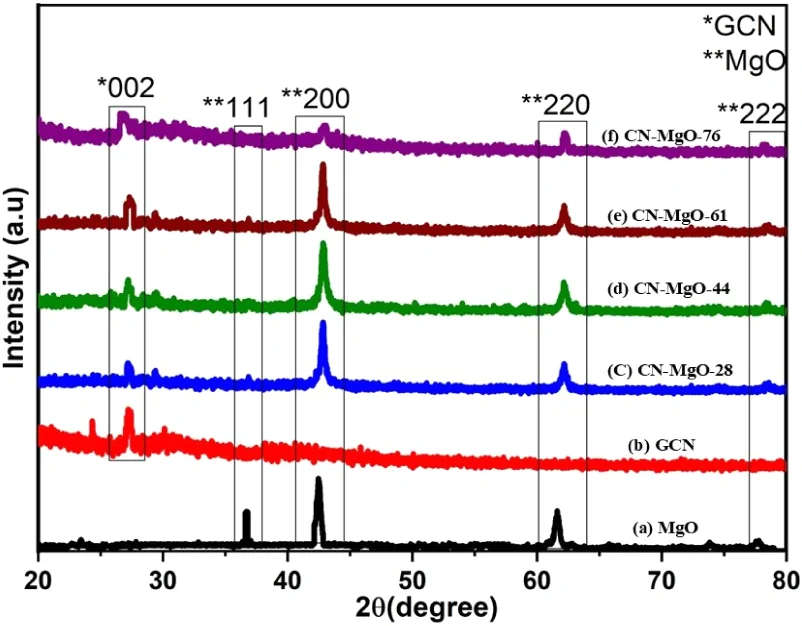
Crystallographic analysis with XRD of (a) MgO, (b) GCN, and (c–f) CN-MgO nanocomposites.

### Morphological Analysis with SEM and TEM

The morphology of the biosynthesized NPs and NCs were studied using SEM as shown in Figure 3 a–f. The pure MgO sample, Figure 3a shows irregularly shaped, agglomerated nanoparticles with a relatively rough and porous surface texture, which is typical of metal oxide nanoparticles.^15,19,40^ In contrast, the GCN sample, Figure 3b exhibits a more layered, sheet-like structure with stacked and wrinkled nanosheets with larger pores than pure MgO, which is characteristic from graphitic carbon nitride morphology.^2,14,17,20^ In the nanocomposites, Figure 3c–f the layered structure of GCN remains dominant. Nevertheless, the porosity in the nanocomposites is visible, which is essential for photocatalytic activity. Overall, the images confirm the successful formation of CN-MgO nanocomposites and demonstrate that irrespective of the MgO content the surface morphology and porosity are retained.^15,23,43,45,48^

**Figure 3:**
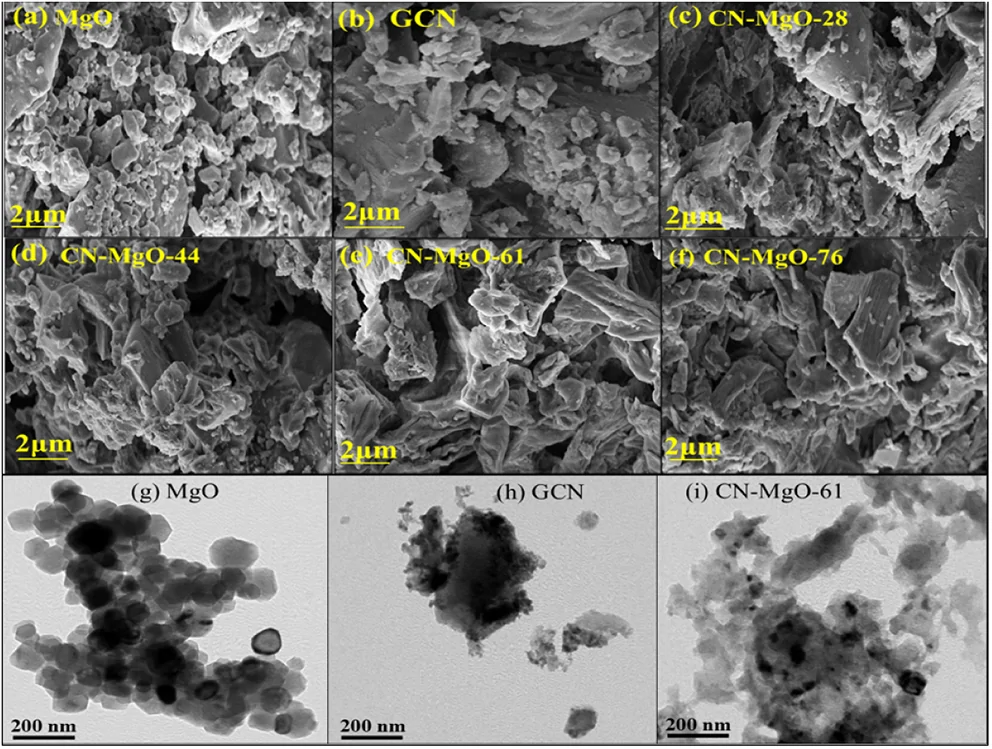
Morphological analysis of the prepared samples: SEM images of (a) MgO, (b) GCN, and (c–f) CN-MgO nanocomposites, and TEM images of (g) MgO, (h) GCN, and (i) CN-MgO-61.

The TEM images show the morphology of pure MgO, pure GCN, and CN-MgO-61 nanocomposite as shown in Figure 3g–i. MgO-NPs Figure 3g exhibit a relatively spherical to slightly irregular shape with strong contrast that highlights their good crystallinity. On the other hand, GCN is aggregated Figure 3h and in the form of layered nanostructure with lower contrast that is typical of layered material such as GCN. In the case of CN-MgO-61 Figure 3i, TEM study reveals a mixed morphology in which darker spots corresponding to MgO-NPs, distributed across the lighter GCN layered matrix. Collectively, these images demonstrate the successful synthesis and integration of MgO-NPs within the GCN layered matrix.^15,23,48^

### Raman Spectroscopy

Raman spectroscopy was employed to probe the structural evolution of GCN following MgO incorporation as shown in Figure 4a–e. All samples, including GCN and CN-MgO nanocomposites exhibit two broad spectral regions centered at 1200–1400 cm^–1^ and 1550–1600 cm^–1^, constituting the D and G bands respectively of disordered and ordered sp^2^ carbon.^46^ Several studies have attributed these D and G bands in GCN and are commonly referred to as, the D-like band (∼1310–1350 cm^–1^) originating from disorder-induced breathing vibrations of aromatic C–N ring structures.^5,46^ Its intensity and position are sensitive to structural defects such as nitrogen vacancies, bond distortions, and local symmetry breaking (Figure S1). The G-like band (∼1560–1595 cm^–1^) corresponds to in-plane stretching of sp^2^-hybridized C–N and C=N linkages and reflects the degree of conjugation and electronic delocalization within the carbon nitride framework.^5^ The presence of these bands confirms the aromatic polymeric phase of g-C_3_N_4_ and offers insights into lattice rearrangement after MgO is introduced into the CN-MgO layered structure.^3,5,46,50,54,55^ The observed Raman features are characteristic of the conjugated carbon–nitrogen structure of graphitic carbon nitride and, together with the existing XRD, FTIR, and XPS results, support the formation of GCN rather than simple carbonized biomass. The Raman spectrum of pure GCN, Figure 4a exhibits characteristic bands at 1325 cm^–1^ (disorder-induced breathing mode of aromatic C–N heterocycles), 1434 cm^–1^ (C–N and C=N stretching), and 1574 cm^–1^ (in-plane stretching of sp^2^ C–N bonds), corresponding to the graphitic carbon nitride framework.^5,54^

**Figure 4:**
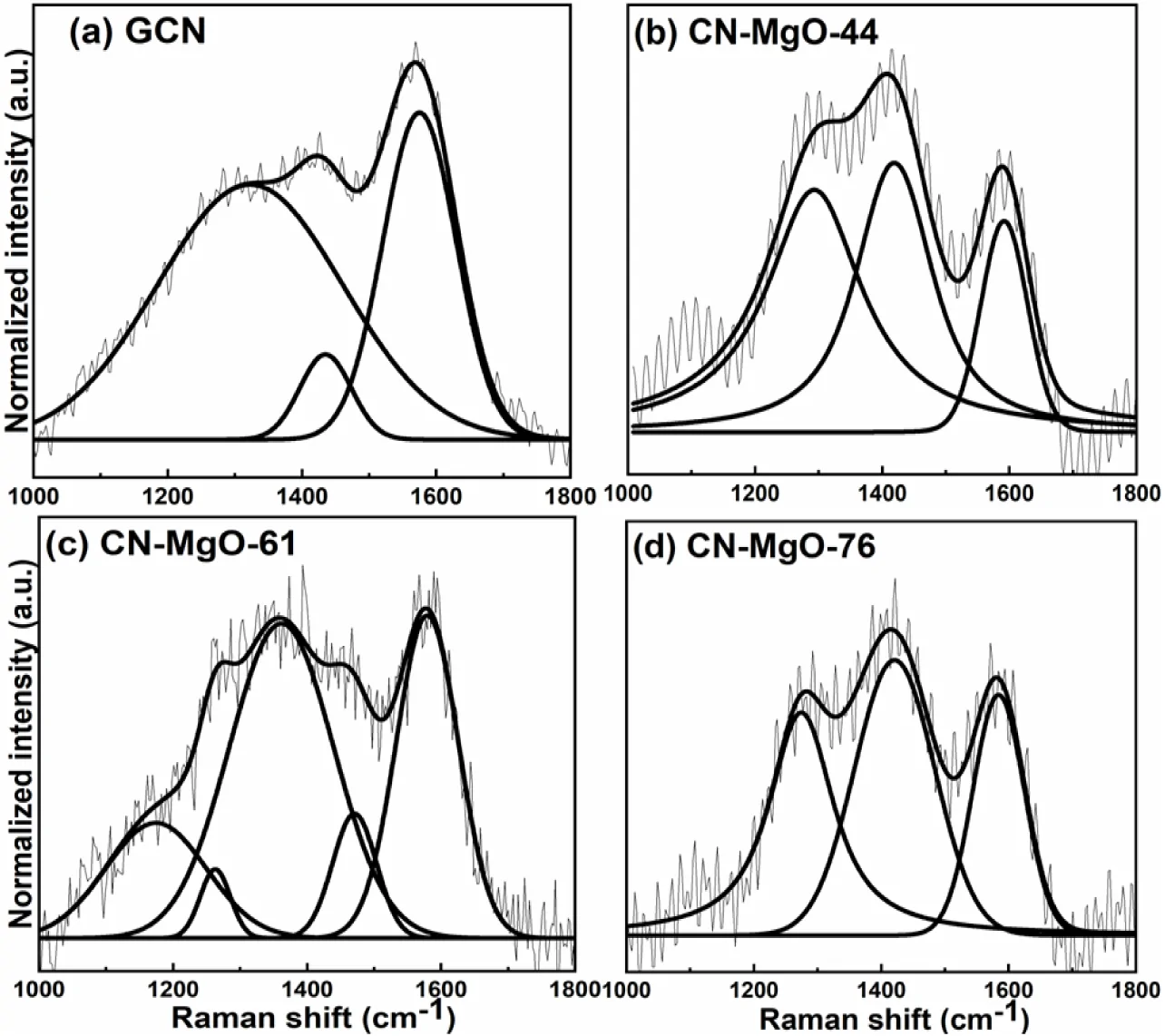
Normalized and deconvoluted Raman spectra of (a) GCN and (b–e) CN-MgO nanocomposites.

Following MgO incorporation, systematic spectral shifts are observed. For the CN-MgO-28 nanocomposite, Figure 4b, a distinct D-like band is not observed suggesting that the structural perturbation introduced at this loading is lower than in pure GCN (Figure S2), which is seconded by the narrower peak width of the G-band compared to the other samples. The MgO nanoparticles most likely passivate the edge defects or the dangling bonds in GCN. More evident structural modification appears in the CN-MgO-44, Figure 4c, where the disorder-associated band shifts from to lower frequencies, accompanied by an upward shift of the graphitic C–N stretching band to higher frequencies. All these shifts also strongly imply the electronic coupling between Mg^2+^ cation and the carbon nitride framework. Consistently, the I_D_/I_G_ ratio increases from 0.81 for pure GCN to 0.88, indicating a higher density of structural defects.^46,50^

The most significant structural evolution is observed for the CN-MgO-61 nanocomposite, Figure 4 d, in which the peak can be deconvoluted into five peaks, indicating the breaking of symmetry and phonon activation by strong interfacial interaction. The band at ∼1359 cm^–1^ reflects disorder-induced breathing vibrations of aromatic C–N heterocycles, while the peak at 1578 cm^–1^ is due to in-plane stretching of sp^2^ -hybridized C–N bonds, confirming that the conjugated network remains nevertheless preserved.^5,50,55,56^ The preservation of associated C–N and C=N stretching vibrations at ∼1472 cm^–1^ confirms once again that the polymeric backbone of GCN remains intact despite substantial lattice distortion.^57^ The D band in the sample corresponds to disordered sp^2^ carbon and indicates the presence of impurities such as C linked to N in the heptazine and triazine networks or the presence of amorphous carbon. These features are characteristic of polymeric carbon nitride and arise from vibrational modes associated with aromatic CN-MgO ring units and the extended sp^2^ -hybridized conjugated network. The presence of ordered sp^2^ carbon in the CN-MgO-61 sample that was also confirmed by XPS, could result from graphitization of the sample when treated at high temperatures. However, further increase of the MgO content in CN-MgO-76, results Figure 4e in a reduced I_D_/I_G_ ratio of 0.81. Overall, Raman study confirms the interaction between MgO and GCN without any obvious structural collapse of the GCN backbone, suggestive of a defect-engineered heterostructure.^50,56^

### Functional Group Analysis Using FTIR

The normalized FTIR absorption spectra of biosynthesized MgO, GCN, and CN-MgO nanocomposites highlight the involvement of phytochemicals in the reduction and stabilization processes of the nanostructures as shown in Figure 5a–f.^12,25,43,44^ In the case of MgO-NPs, Figure 5a, the bands at 627 cm^–1^, 665 cm^–1^, 943 cm^–1^, and 1047 cm^–1^, indicate the formation of Mg–O nanoparticles. The presence of these characteristic Mg–O bands indicates the formation of a metal–oxygen framework and verifies the conversion of the precursor into crystalline MgO nanostructures. In particular, the lower wavenumber bands are attributed to lattice vibrations of Mg–O bonds, which are commonly reported for MgO-based nanomaterials in the literature. The broad band in the 1330–1600 cm^–1^ range are due to the presence of carbonyl groups (C=O). The peak at 3698 cm^–1^ confirms the presence of hydroxyl (−OH) groups from adsorbed water molecules and polyphenolic compounds that act as stabilizing agents.^16,18,19,45^ The GCN sample exhibits, Figure 5b a peak at 790 cm^–1^ that can be attributed to triazine ring vibrations. Peaks at 1320 cm^–1^ and 1482 cm^–1^ are associated with C–N and C=N stretching that are typical features of graphitic carbon nitride. The bands at 1608 cm^–1^ and 1691 cm^–1^ correspond to C=N and C=O stretching, likely due to oxidation of GCN. The peak at 2999 cm^–1^ is ascribed to C–H stretching from remaining organic groups.^4,18^ Overall, the presence of these characteristic vibrational bands confirms the successful formation of the polymeric graphitic carbon nitride framework with conjugated nitrogen-containing heterocyclic units. In particular, the distinct triazine—related vibration at ∼790 cm^–1^ serves as a fingerprint feature confirming GCN formation.^15,16^ In the case of CN-MgO nanocomposites, Figure 5c–f exhibits noticeable variations in both peak intensity and peak position compared with pure GCN, indicating strong interfacial interactions between MgO and the GCN framework. The broad absorption band in the 3200–3700 cm^–1^ region becomes more pronounced in the composites, suggesting enhanced hydrogen-bonding interactions between surface −OH/Mg–OH groups of MgO and residual −NH groups of GCN. In addition, slight shifts and intensity changes observed for the C–N/C=N stretching vibrations (1320–1691 cm^–1^) indicate electronic interaction and possible coordination between Mg^2+^ ions and nitrogen sites of the triazine unit.^27,44^ Furthermore, the gradual modification of the characteristic triazine-related vibration near 790 cm^–1^ with increasing MgO concentration suggests partial distortion/reorganization of the GCN aromatic framework due to MgO incorporation. The Mg–O-related bands in the lower wavenumber region also become more distinct in CN-MgO-61 and CN-MgO-76, confirming the increased contribution of MgO within the composite structure. These spectral variations collectively indicate the successful formation of an interracially coupled CN-MgO heterostructure rather than a simple physical mixture. Such interfacial interactions can facilitate improved charge transfer, enhanced surface activity, and stronger anchoring between both components, thereby promoting the synergistic photocatalytic behavior of the CN-MgO nanocomposites.^40,46–49^

**Figure 5:**
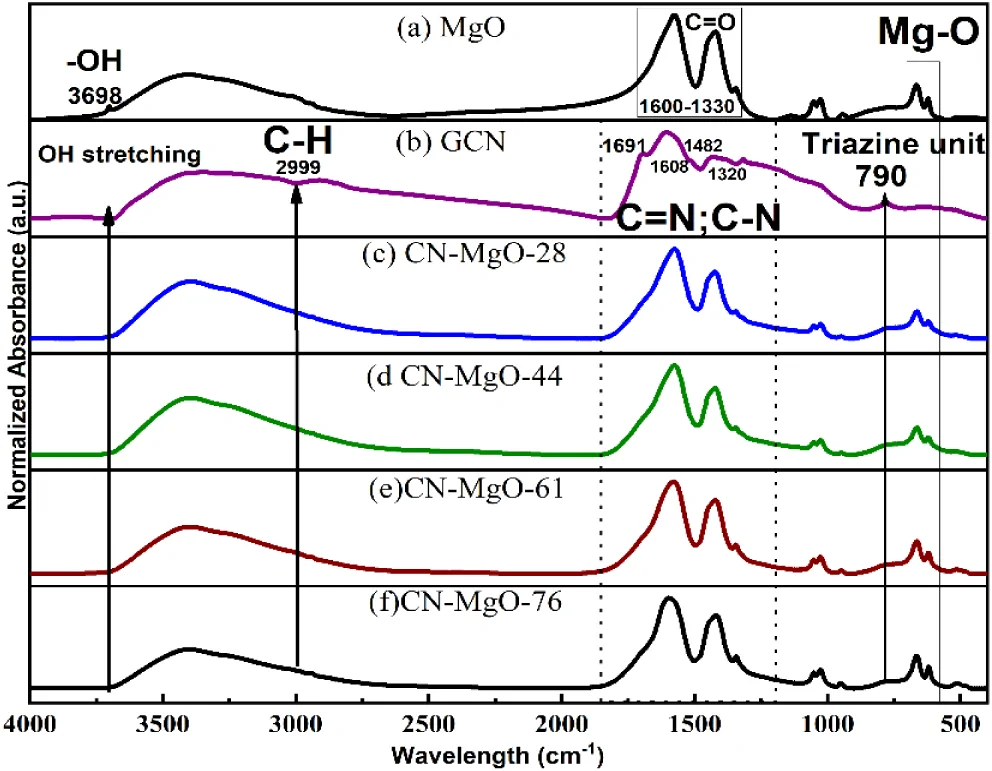
Normalized FTIR spectra in absorbance mode of (a) MgO, (b) GCN, and (c–f) CN-MgO nanocomposites.

### Surface Chemical Analysis with XPS

XPS was employed to study the composition, chemical states, and interfacial electronic interactions of pure GCN and CN-MgO-61 and CN-MgO-76 nanocomposites as shown in Figure 6i,a–c. The high-resolution C 1s spectra of pure GCN can be deconvoluted into three components located at 284.66 eV (C–C/C=C), 285.59 eV (C–N/C–H), and 286.38 eV (C–O). The C–O contribution is mainly attributed to adventitious carbon. The relatively intense C–C/C=C component at ∼283.2 eV suggests the presence of sp^2^ -hybridized carbon domains, which in this case is attributed to partial carbonization of organic constituents from the biowaste precursor, leading to the formation of graphitic or graphitic-like carbon species in addition to the polymeric GCN framework.^9,43,46^ For the CN-MgO-61 sample, the main C–C/C=C peak shifts slightly toward a lower binding energy, accompanied by small shifts in the C–N/C–H and C–O components, indicating electronic interaction between MgO and the GCN framework. A binding energy peak is visible at ∼283.28 eV, corresponding to sp^2^ -hybridized carbon in N–C=N units,^9,35^ as also observed in the Raman spectrum of this sample. Such graphitic or amorphous carbon phases may be present alongside the GCN framework in this system.^2,46^ A distinct high-binding-energy peak at ∼289.91 eV is assigned to interfacial C–O–Mg bonding, providing direct evidence for chemical interactions between CN-MgO and MgO, consistent with the FT-IR results. Compared with CN-MgO-61, the CN-MgO-76 composite exhibits lower relative intensities of the C–C/C=C and C–O–Mg components, indicating a diminished contribution of carbon-containing species at the surface. This reduction is likely due to a shielding effect arising from the higher MgO nanoparticle content and their probable agglomeration.

**Figure 6:**
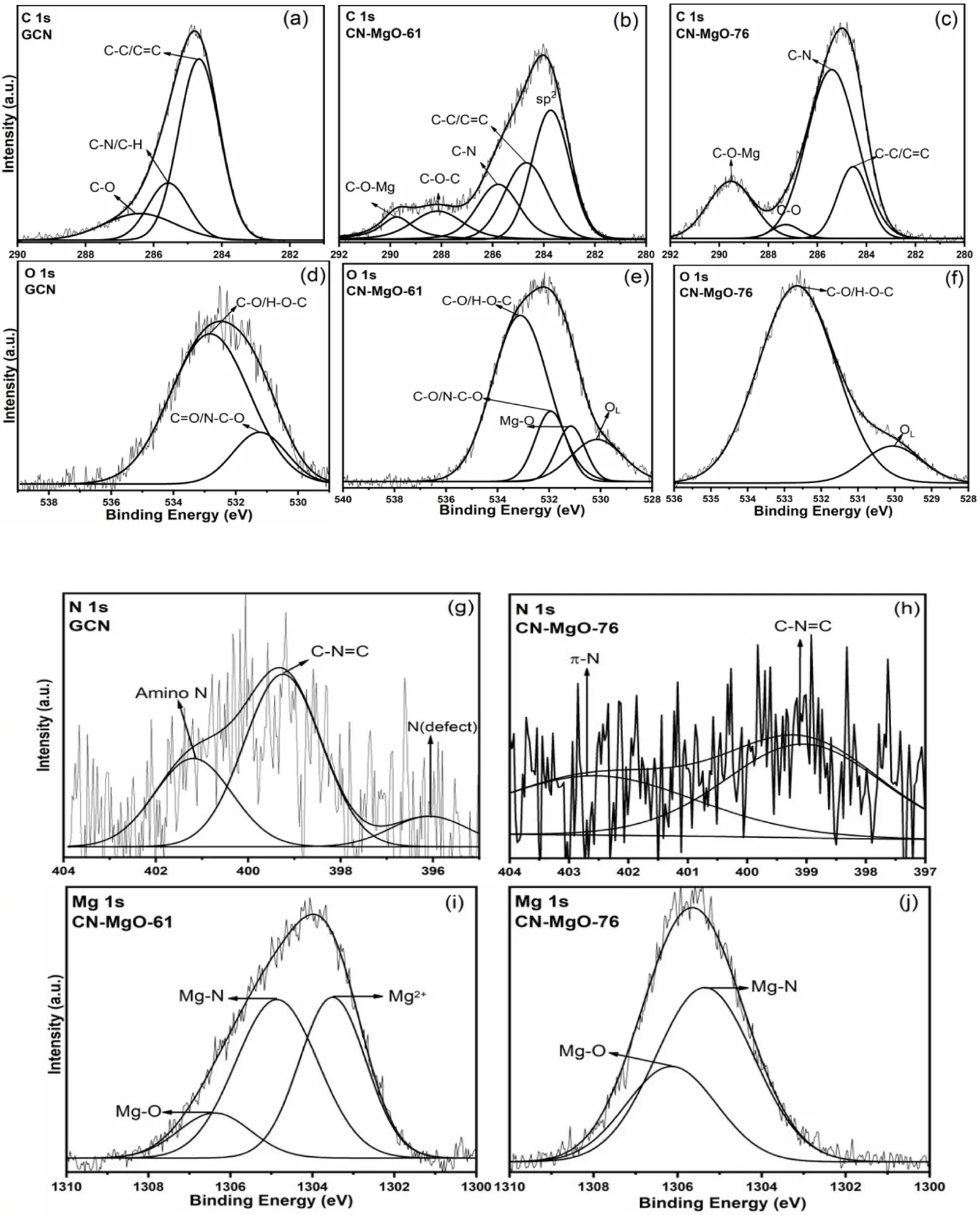
**(i)** high-resolution XPS spectra of C 1s (a–c) and O 1s (d–f) of GCN, CN-MgO-61, and CN-MgO-76. **(ii)** High-resolution XPS spectra of N 1s (g, h) for GCN and CN-MgO-76, and Mg 1s (i, j) for CN-MgO-61 and CN-MgO-76 nanocomposites.

The O 1s spectra of pure GCN Figure 6i, d–f shows two components at 531.25 and 532.85 eV, attributed to oxygen-containing surface species such as C=O and N–C–O functionalities, as well as surface hydroxyl groups or adsorbed oxygen species. These oxygen contributions originate from the biowaste-derived precursor and the thermal treatment performed under ambient air atmosphere, and are consistent with the FTIR results.^46^ For the CN-MgO-61 composite, the O 1s spectrum broadens and can be deconvoluted into multiple components. A low-binding-energy peak at 530.15 eV is assigned to oxygen species associated with MgO, typically attributed to lattice O^2 –^ in bulk-like MgO. Additional components in the 531–532 eV range are attributed to oxygen species in modified chemical environments, which may include surface or interfacial Mg–O species as well as oxygen-containing groups associated with the GCN matrix. A higher-binding-energy component at 533.21 eV is assigned to surface hydroxyl groups or adsorbed oxygen species. In the CN-MgO-76 composite, similar O 1s features are observed.

High-resolution of N 1s spectra are shown in Figure 6ii,g–h. Pure GCN exhibits three components at 399.25 eV, corresponding to sp^2^ -hybridized nitrogen in C–N=C units at 401.20 eV attributed to amino nitrogen, and a low-binding-energy feature at 396.05 eV assigned to electron-rich, defect-associated nitrogen species (N_(defect)_). These defect-related nitrogen species are associated with structural imperfections such as nitrogen vacancies, under-coordinated edge nitrogen atoms, and locally distorted C–N environments, which are typical for biomass-derived and air-treated GCN. The N 1s signal of the CN-MgO-61 composite sample was too weak for reliable deconvolution; however, analysis of the corresponding C 1s spectrum confirms the presence of nitrogen-containing species. This observation is further supported by the results of Raman and FTIR spectroscopy. For the CN-MgO-76 composite, two N 1s components appear at 399.08 and 402.57 eV, corresponding to *sp* ^2^ nitrogen in C–N=C units and π-electron-delocalized nitrogen species,^4,43^ respectively. The latter is slightly shifted to a higher binding energy, suggesting passivation or modification of surface nitrogen sites upon interaction with MgO. This indicates that although the GCN framework remains structurally intact, its electronic structure is modified by interaction with MgO, consistent with the formation of an interfacial Mg–O–N bond. The Mg 1s spectrum corroborates these findings as shown in Figure 6ii, i,j. The CN-MgO-61 composite exhibits Mg^2+^ peaks at 1303.52 and 1304.94 eV, corresponding to Mg species in different chemical environments, while a higher binding energy peak at 1306.43 eV is assigned to Mg–O bonding, confirming the presence of MgO well integrated within the composite. In contrast, the CN-MgO-76 composite shows Mg 1s peak shifted to higher binding energies, consistent with a greater contribution from bulk-like MgO. Overall, the systematic binding-energy shifts and intensity variations in the C 1s, N 1s, and O 1s spectra, together with the relative intensities, indicate strong interfacial electronic interactions between MgO and GCN. The CN-MgO-61 composite shows richer spectral features, consistent with a higher contribution of exposed carbonaceous domains, whereas the CN-MgO-76 sample is dominated by MgO-related signals due to increased surface coverage, with both samples confirming the formation of a chemically coupled CN-MgO heterostructure.

### Optical Analysis Using UV–Vis Absorption and Photoluminescence Spectroscopy

The optical properties of OP mediated MgO nanoparticles, GCN, and CN-MgO nanocomposites were studied using a UV–vis spectrophotometer within the wavelength range of 200 to 1100 nm. Tauc plots were generated to evaluate the direct band gap using the formula αhν^2^=(hν-*Eg*) as shown in Figure 7a–f, and indirect transition using the formula αhν^1/2^=*A*(hν-*Eg*) Figure 7a–f insets. OP mediated MgO nanoparticles Figure 7a, has direct and indirect optical band gaps of 4.25 and 4.13 eV respectively.^41,55^ The band gap is lower than for bulk MgO that possesses a direct band gap of about 7.3–7.8 eV, due to its ionic Mg^2+^ and O^2 –^ bonding.^41,43,46^ The valence band of MgO is mainly composed of oxygen 2p orbitals, which gives MgO strong oxidizing ability due to the high oxidation potential of its photogenerated h^+^ which plays an important role in dye oxidation during photocatalysis. The conduction band is mainly derived from Mg 3s orbitals.^41,46^ MgO-nanoparticles harbor surface defects and oxygen vacancies that introduce localized states within the band gap, facilitating optical transitions in the visible region. Pure GCN has a direct band gap of 2.7–3.3 eV, mainly from π–π* transitions between nitrogen 2p-derived valence bands and carbon 2p-derived conduction bands.^8,10^ However, in this study, GCN has a direct optical band gap at 3.26 eV and an indirect optical band gap of ∼3.1 eV. In Figure 7b, several lower energy indirect transitions at 1.7–3.1 eV indicative of defect-related transitions, such as nitrogen or carbon-related vacancies resulting from an incomplete polymerization of triazine unit or structural disorder within the GCN layers.^32,46^ These sub-bandgap transitions, including n-π* excitations from nitrogen lone pairs, allow absorption of lower-energy photons.^39^

**Figure 7:**
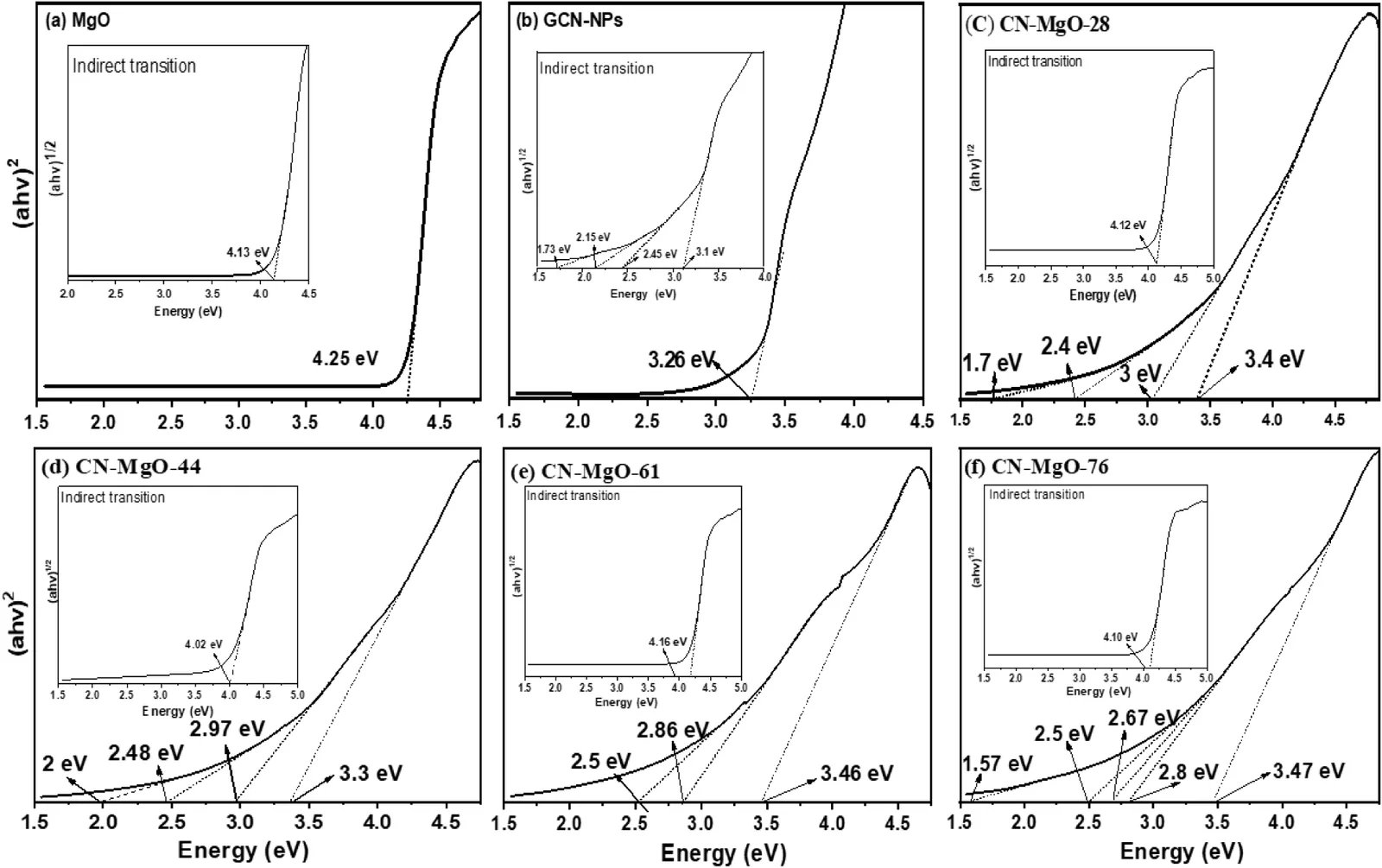
Tauc plot of (a) MgO, (b) GCN, and (c–f) CN-MgO nanocomposites.

The sub-bandgap transitions do not manifest as distinct features in the Tauc plot constructed for direct transitions in both MgO and GCN, indicating that the optical absorption is dominated by the fundamental band-to-band transitions for GCN, which also exhibits the highest intensity. The Tauc plot for indirect transitions in MgO likewise does not reveal any clear defect-related absorption features. Moreover, the direct and indirect optical band gaps of MgO are very close in energy, suggesting that phonon-assisted indirect transitions can occur with comparable probability in this material. In the CN-MgO-28, Figure 7c, the absorption at 3.4 eV represents the new optical band gap of MgO that has been reduced due to changes to its surface defects on combining with GCN. Here, 3 eV corresponds to the optical bandgap of GCN and 2.4 eV, and is likely due to the bandgap states in both MgO (F centers) and GCN.^41,42,49,55^ These states lie just below the conduction band of GCN. Additionally, the 1.7 eV transition is attributed to midgap defects that are commonly associated with Mg point defects or other deep trap sites introduced at the CN-MgO interface. The indirect band gap for this material is dominated by the MgO indirect transition of 4.12 eV. Similarly, for the other CN-MgO-28, -44, -61, and -76 nanocomposites, Figure 7d–f the indirect bandgaps show a unique absorption exceeding 4 eV and correspond to MgO indirect transitions. In the direct transitions, the MgO optical band gap is reduced, and lies between 3.3 and 3.4 eV due to surface defects and changes in F centers. The GCN optical band gaps are between 2.87 and 2.96 eV. All these nanocomposites show similar defect level absorptions between 2 and 2.5 eV. The proposed band alignment is based on literature reports of band-edge positions and on the optical properties studied in the present work. Since no direct measurements of the conduction- and valence-band positions were performed, the charge-transfer is only a qualitative model.

### Photoluminescence Spectroscopy

Photoluminescence spectroscopy was employed to investigate the excitonic recombination behavior in GCN, MgO, and CN-MgO nanocomposites as shown in Figure 8a–f. This technique is particularly helpful as it elucidates the optical activity of the defect states both within the volume and at the surface of the nanomaterial (Figure S3). The blue emission centered at ∼394 nm (≈3.15 eV), Figure 8a is attributed to the near-band-edge radiative recombination in GCN, associated with π*−π and/or π*–n transitions of the conjugated C–N framework.^10,32^ This emission band can be deconvoluted into two additional components located at approximately 404 and 410 nm. These features are assigned to shallow donor states associated with nitrogen vacancies and under-coordinated framework carbon sites created by nitrogen loss.^39,55^ Such donor states are typically located about 0.1–0.4 eV below the conduction band minimum and are responsible for the intrinsic n-type character of GCN.^58^ A weak visible emission is also observed in the range of 500–575 nm, with a maximum at ∼530 nm. This band is attributed to deep localized states associated with strong structural relaxation around nitrogen-vacancy-related defects.^10,32,39^ Nevertheless, this deep-level emission remains relatively weak compared to the near-band-edge emission arising from the π*−π and π*–n transitions of GCN. In the case of MgO, Figure 8b, the emission profile appears rather similar, with a dominant near-UV emission centered at ∼397 nm and weaker components at approximately 406 and 418 nm. However, the physical origin of these emissions in MgO is different from GCN. Considering the optical band gap of MgO extracted from UV–vis measurements (∼4.2 eV), the emission at ∼397 nm (3.15 eV) cannot be attributed to band-to-band recombination. Instead, it is assigned to defect-related luminescence originating from F^+^ centers, i.e., singly ionized oxygen vacancies (V_O_^+^).^40,41,49,55^ The weaker green emission around ∼ 540 nm is attributed to F centers, corresponding to neutral oxygen vacancies (V_O_) containing two trapped electrons. The relatively low intensity of the F-center emission suggests that these defects mainly act as trapping or nonradiative recombination centers, meaning additional nonradiative decay pathways are active in MgO. The optical absorption spectra of MgO do not show distinct absorption edges associated with these defect centers at 3.1 eV and between 2.3 eV–2.5 eV, indicating that the absorption cross-section of the vacancy-related states is much weaker than that of the main optical transition. It also indicates that the charge transfer to these defect states occurs via relaxation of conduction band electrons via radiative or nonradiative pathways, rather than direct optical excitation. A similar overall emission profile is observed for the CN-MgO nanocomposites, Figure 8c–f. However, the intensity of the ∼530 nm emission decreases progressively with increasing MgO content, suggesting that these deep trap states become less optically active in the composites, even though their presence can still be inferred from the absorption spectra.^15,23,43,48,59^

**Figure 8:**
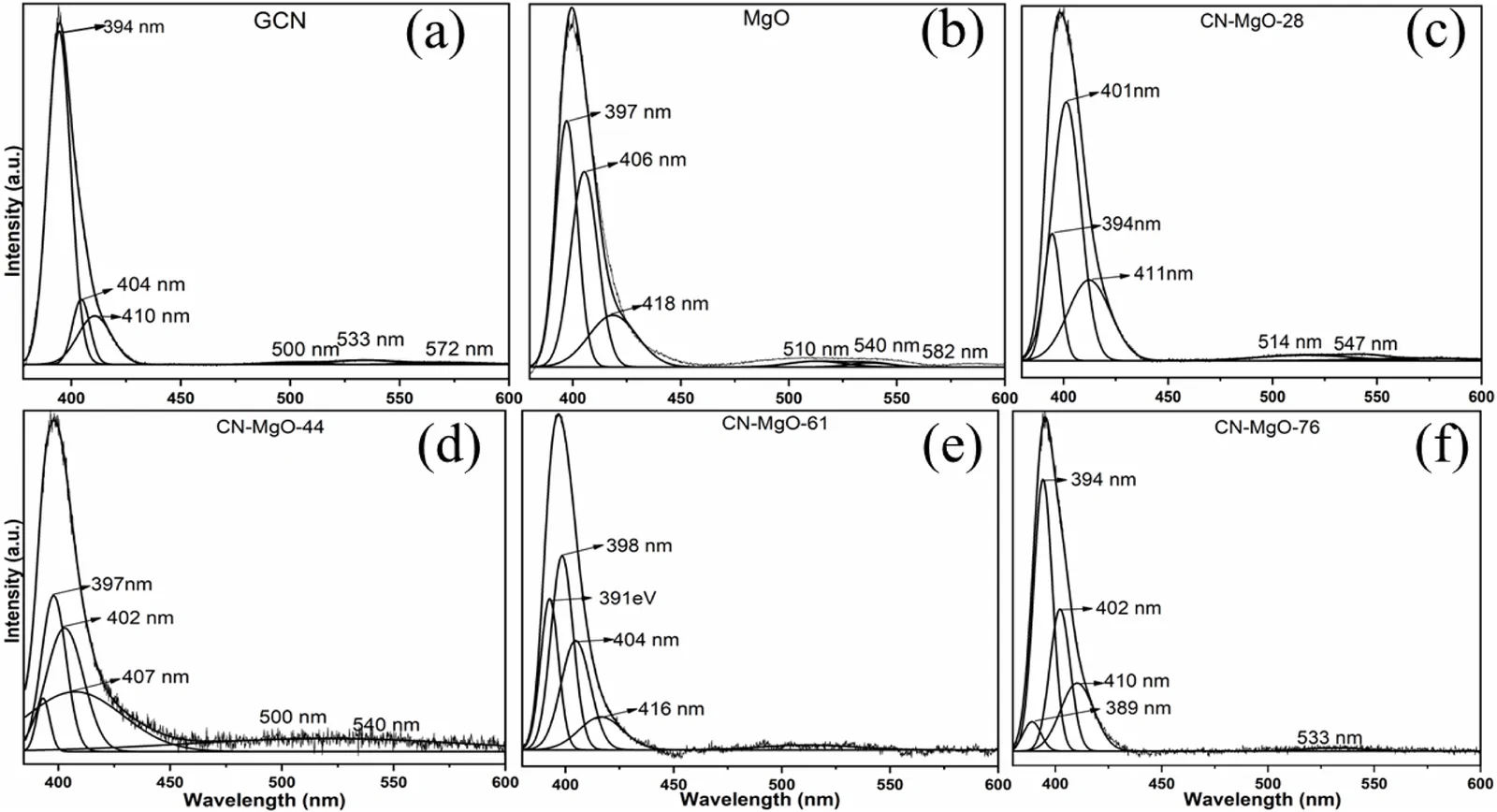
Deconvoluted photoluminescence spectra of (a) GCN, (b) MgO, and (c–f) CN-MgO nanocomposites.

### BET Analysis

The adsorption of N_2_ gas in the relative pressure range (P/P_0_ ≤ 0.25) was used to determine the monolayer adsorption capacity according to the BET model as shown in Figure 9a–d. While the corresponding textural parameters are summarized in Table 3. The measured pore volumes were negligible (Vp ≈ 0), indicating a limited contribution from accessible porosity. The specific surface area of pure GCN (2.4 m^2^·g^–1^) was higher than that of CN-MgO-61 (1.3 m^2^·g^–1^), suggesting that incorporation of MgO reduced the accessible surface area, possibly due to particle aggregation or partial coverage of the GCN surface by MgO nanoparticles.^15,18^ Nevertheless, the average pore size, monolayer adsorption capacity (Qm), and BET constant (CBET) were similar for both samples, indicating comparable adsorption affinities toward N_2_. It should be noted that BET measurements are performed on dry powders, whereas the photocatalytic experiments were conducted in aqueous suspensions. Therefore, the BET surface area alone cannot fully explain the observed photocatalytic performance.^4,6,14^ Despite its lower measured surface area, CN-MgO-61 exhibited significantly enhanced photocatalytic activity compared with pure GCN. This behavior suggests that improved charge separation, defect-mediated charge transfer, and interfacial interactions between MgO and GCN play a more important role than surface area in determining photocatalytic efficiency. In addition, the presence of MgO may improve the dispersion stability of the composite in aqueous media.^15,42,48^

**Table 3:** BET Parameters of the GCN and CN-MgO-61

Sample	S_BET_ (m^2^/g)	C_BET_	Q_m_ (cm^3^/g)	V_p_ (cm^3^/g)	Pore size (nm)
**GCN**	2.4	20.7	0.5	0	1.7
CN-MgO-61	1.3	21.9	0.3	0	1.5

**Figure 9:**
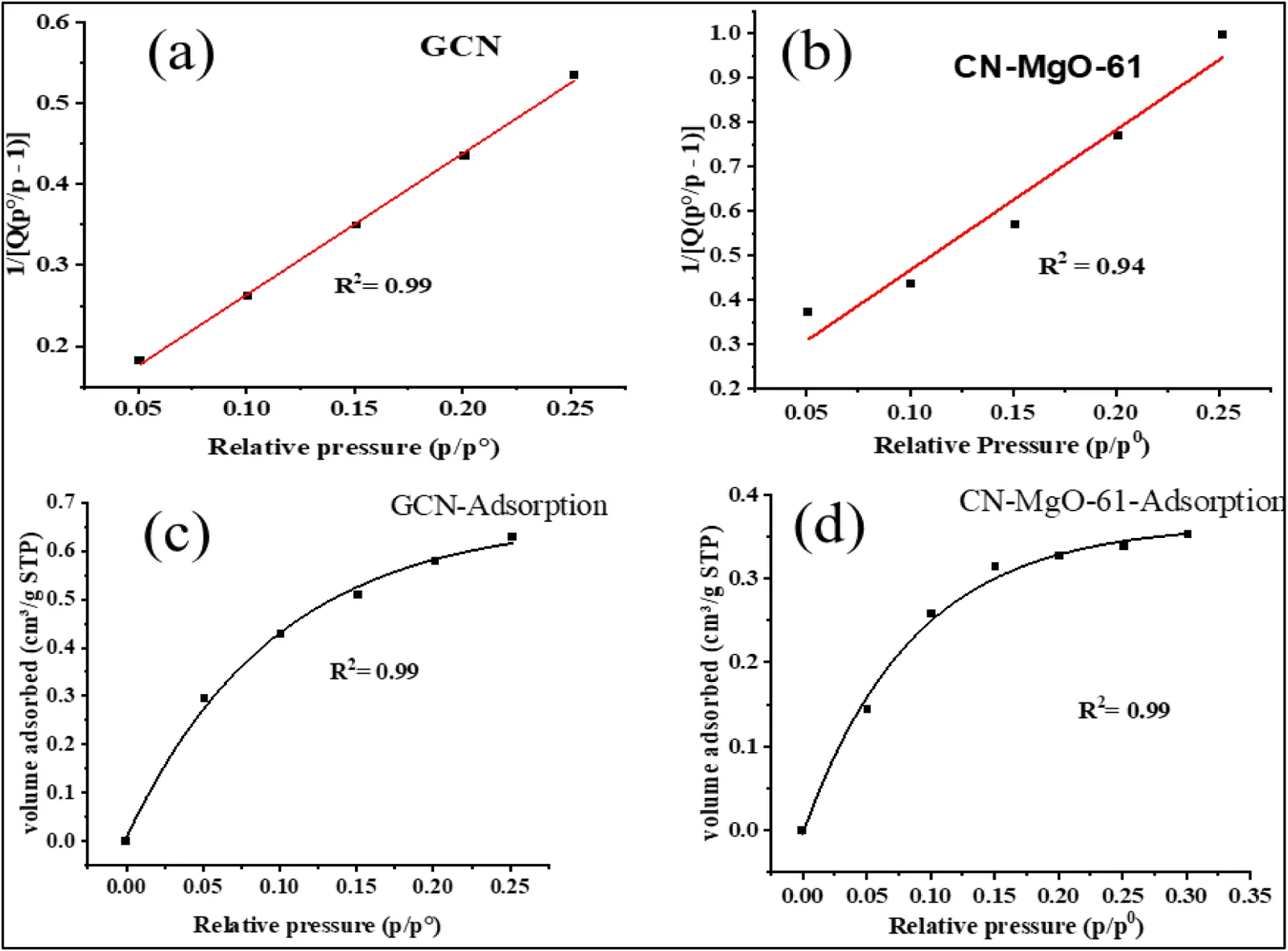
BET plots for N_2_ adsorption and their isotherms on GCN (a) and (c), and CN-MgO-61 (b) and (d).

### Cytotoxicity Evaluation

The cytotoxicity of these nanomaterials was evaluated in order to ensure biosafety and compatibility with living cells. (i) Cell treatment; 24-h postseeding cells were treated with 50 μg/mL of MgO, GCN, and CN-MgO nanocomposites, all dissolved in Dimethyl Sulfoxide (DMSO), with each treatment performed in triplicate. (ii) Incubation; the treated cells were incubated for 24-h under standard culture condition to allow interaction between the nanoparticles and the cells. (iii) Viability staining after the incubation period, the cells were stained with trypan blue dye in order to evaluate membrane integrity. (iv) Assessment viability; viable cells exclude trypan blue conserving the membrane integrity, while nonviable cells absorb the dye, allowing for differentiation and counting under a microscope as shown in Figure 10a (i), b (i), c (i), d (i), e (i), f (i), and g (i) before incubation) and (**a (ii), b (ii), c (ii), d (ii), e (ii), f (ii),** and **g (ii)** after incubation). The control group, with 90–95% living cells, sets a benchmark for cell viability in the absence of nanoparticle treatment, indicating healthy cell growth conditions as shown in Figure 10a (ii) and h. The cytotoxicity results indicate that the synthesized MgO-NPs exhibit 80–90% cell viability at a high dose of 50 μg/mL, suggesting relatively lower cytotoxicity, while still indicating some degree of cellular stress. Similar observations have been reported in previous studies,^59–62^ where MgO nanoparticles showed a dose-dependent decrease in cell viability, with values decreasing to about 80% at a concentration of 10 μg/mL and further declining to 20% for a higher concentration of 140 μg/mL, mainly due to oxidative stress and nanoparticle-cell interactions.^61,62^

**Figure 10:**
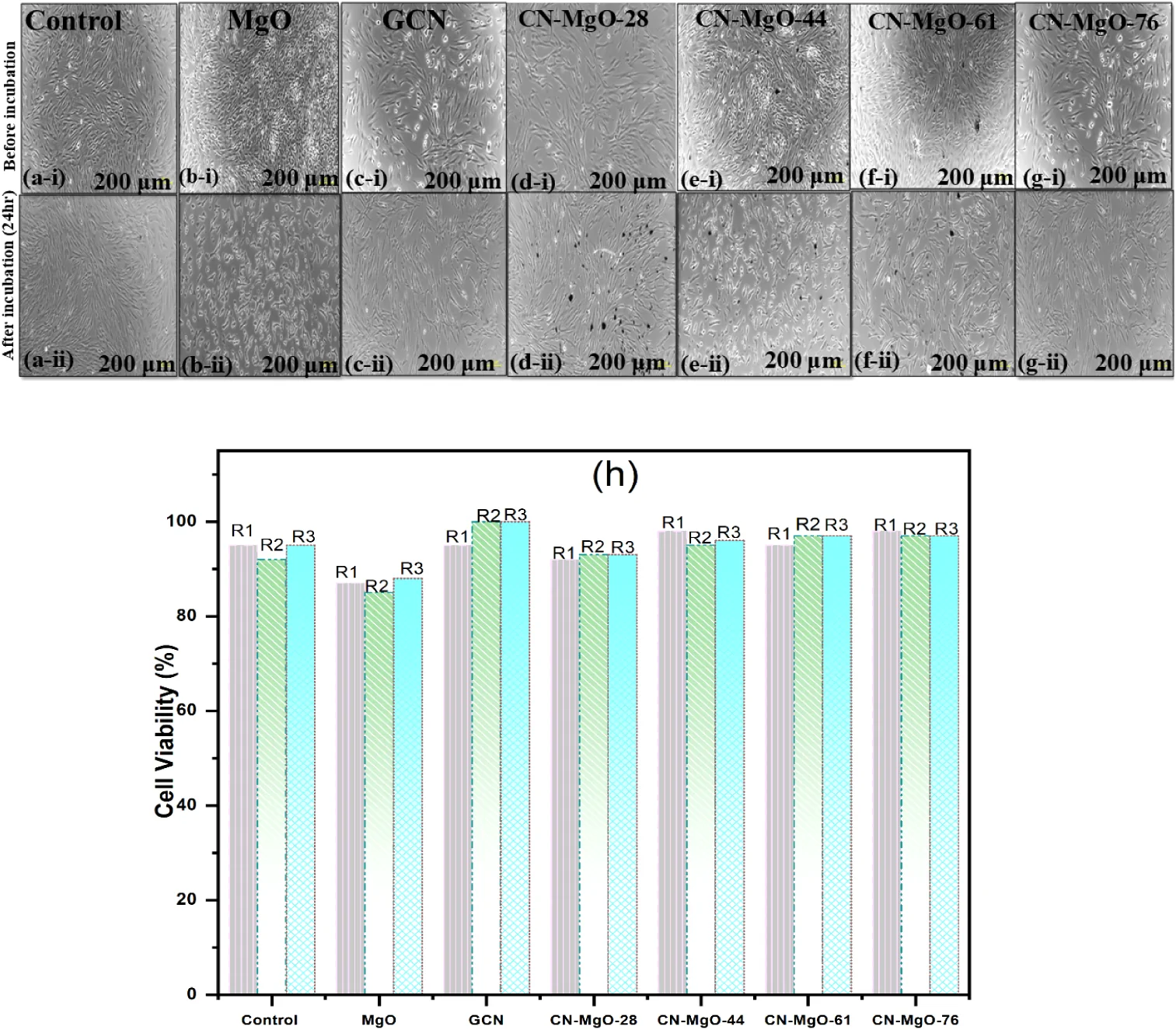
Microscopy images of cytotoxicity potential of biosynthesized NPs and nanocomposites; before incubation-a­(i) control, b­(i) MgO, c­(i) GCN, and d­(i)–g­(i) CN-MgO nanocomposites; after incubation a­(ii) control, b­(ii) MgO, c­(ii) GCN, and d­(ii)–g­(ii) CN-MgO nanocomposites; h-showing cell viability % after 24-h incubation), R1, R2, and R3 state for readings.

In contrast, in this study, the synthesized GCN demonstrated 95–100% cell viability, indicating excellent cytocompatibility for a relatively high concentration of 50 μg/mL. Carbon-based nanomaterials such as GCN are widely regarded as biocompatible due to their chemically stable layered structure and relatively inert surface characteristics. For instance, Drdanová et al.^63^ reported that exhibits low intrinsic toxicity and maintains high cell viability often approching 100% across a range of biological models, attributing this to its metal-free composition and chemical stability. Similarly, Alonso et al.^64^ highlighted that GCN-based systems show minimal cytotoxic effects 85% to 95% in cancer-related biological environments, largely due to their controlled surface reactivity and low induction of oxidative stress. Furthermore, Suner et al.^65^ demonstrated that GCN composites used in bioimaging applications maintain high cytocompatibility ≥90%, as their stable framework prevents harmful interactions with cellular components. In fact, nanomaterials with stable surfaces and reduced aggregation tendencies exhibit lower cellular internalization and diminished activation of stress pathways, which directly supports the high viability observed for GCN.^66^ In addition, GCN-based drug delivery systems enable controlled interactions with cells, minimizing toxicity through regulated surface chemistry and reduced nonspecific uptake.^67^ Furthermore, incorporation of metal species into GCN can modulate cytotoxicity by stabilizing reactive sites and controlling metal ion release.^68^ Similarly, in this study, the CN-MgO nanocomposites exhibited 94–100% cell viability as seen in Figure 10a–h, which is significantly higher than that observed for pure MgO nanoparticles (80–90%). This improvement suggests that incorporation of MgO nanoparticles within the GCN matrix reduces direct nanoparticle–cell interactions. In the CN-MgO nanocomposite structure, MgO nanoparticles are anchored within the layered GCN framework, enhancing structural stability and preventing MgO nanoparticle dispersion. Such stabilization can limit excessive nanoparticle internalization and reduce oxidative stress-induced cytotoxicity.

A comparable synergistic effect has been reported by Farhang et al.,^69^ where ZnFe_2_O_4_-decorated g-C_3_N_4_/GO composites showed improved biocompatibility relative to pristine metal oxide nanoparticles. The authors attributed this to the confinement of ZnFe_2_O_4_ within the g-C_3_N_4_/GO matrix, which reduced nanoparticle aggregation, limited direct membrane damage, and suppressed ROS overproduction. Similarly, previous studies on g-C_3_N_4_-based hybrid systems^68^ have demonstrated that embedding metal species into the g-C_3_N_4_ framework regulate surface reactivity, which in turn, enables a controlled ion release, thereby mitigating cytotoxic effects. Consequently, the enhanced cytocompatibility observed in CN-MgO nanocomposites can be directly correlated with matrix-assisted nanoparticle stabilization, reduced cellular uptake, and attenuation of ROS-mediated damage-mechanisms that are consistent with previously reported GCN-based composite systems.^68,69^

### Photocatalytic Performance of MgO, GCN, and CN-MgO Nanocomposites

The photocatalytic removal of SO dye was tested with MgO, GCN, and CN-MgO-28, CN-MgO-44, CN-MgO-61, and CN-MgO-76 nanocomposites a solar simulator for a period of 120 min. To evaluate the possible contribution of dye photobleaching, control experiments were performed under identical solar simulator irradiation in the absence of the photocatalyst. Negligible degradation of SO was observed Figure 11a, indicating that direct dye photobleaching contributed negligibly to the overall removal process. Consequently, the observed degradation can be attributed predominantly to the photocatalytic activity of the synthesized CN-MgO materials under visible-light irradiation rather than to self-degradation of the dye. The dye degradation results were obtained through UV–vis absorbance measurements in the range of 200 nm–800 nm. The photocatalytic properties of all biosynthesized samples toward SO degradation is shown in Figure 12a,b and Figure S4. The exposure of SO without catalyst, Figure 12a under the solar simulator does not show any photobleaching, highlighting the high stability of the dye under visible light exposure. The strong absorption band characteristic of SO is observed at approximately 519 nm and was used to assess SO removal from the solution. In addition to the main visible absorption peak, another absorption band is also visible at approximately 276 nm, which is attributed to π–π* transitions associated with aromatic ring structures, specifically the conjugated aromatic phenazinium chromophore of the dye containing π delocalized electrons as shown in Figure 11a.^60–62^ During photocatalytic degradation, the chromophoric group is typically attacked first by reactive oxygen species (ROS), leading to a rapid decrease in the 519 nm peak that corresponds to the chromophore degradation. However, the intermediate degradation products continue to absorb in the UV region the 260–280 nm region. Therefore, the gradual decrease of the 276 nm absorption indicates progressive breakdown of the aromatic ring structures following chromophore bond breakage. This confirms a clear dye degradation mechanism giving rise to degradation products or mineralization rather than only decolorization of the dye.^61^ Also, for the pure MgO and GCN samples, the completely breakdown of these chromophores was not observed and these peaks remained sharp with only a loss in intensity. For all samples the degradation efficiencies with SO removal was 43%, 45%, 79%, 84%, 89%, and 83% for GCN-NPs, MgO-NPs, and CN-MgO-28, CN-MgO-44, CN-MgO-61, and CN-MgO-76 nanocomposites, respectively. All the nanocomposites display enhanced photocatalytic activity with efficiencies ranging from 80%–89% compared to pure MgO and GCN. This suggests that MgO and GCN alone exhibit lower photocatalytic properties under sun light with an average removal of SO (around 44–45%), whereas when combined, a synergistic degradation process occurs. The increase in dye degradation of the nanocomposites can be attributed to the reduced optical activity of the defect states, which allows more efficient charge separation as observed in their PL spectra. The active sites on the surface of the nanoparticles therefore trap charges as observed in the UV–vis absorption spectra. These charges are then transferred to the dye molecule and for the production of ROS. The highest dye degradation activity of 89%, observed with CN-MgO-61 suggests that such a ratio corresponds to an optimal MgO dispersion and synergistic effects with the GCN matrix. When the MgO content is slightly higher as in CN-MgO-76, the photocatalytic efficiency decreases slightly but remains very close to the CN-MgO-61 sample. The photocatalytic degradation kinetics of SO dye were further analyzed using the Langmuir–Hinshelwood pseudo-first-order kinetic model, and the corresponding apparent rate constants (k) were calculated from the slope of the linear plots of ln­(C_0_/C) versus irradiation time as shown in Figure 11c. The obtained rate constants are summarized in Table 4. The values of k were 0.00347 min^–1^ and 0.0044 min^–1^ for GCN-NPs and MgO-NPs, respectively, indicating relatively slow degradation kinetics when the individual materials were used as photocatalysts. In contrast, the CN-MgO-28, CN-MgO-44, CN-MgO-61, and CN-MgO-76 nanocomposites exhibited significantly higher rate constants ranging from 0.01128 to 0.01609 min^–1^, demonstrating a substantial enhancement in photocatalytic activity upon coupling MgO with the GCN matrix. Here, CN-MgO-61 showed the highest rate constant (0.01609 min^–1^), which is approximately 4.6 times higher than GCN and about 3.7 times higher than MgO-NPs, confirming the superior catalytic performance of this nanocomposite. This enhancement indicates that the heterojunction formed between MgO and GCN significantly improves charge separation efficiency and suppression of electron–hole recombination, which leads to a more effective generation of reactive oxygen species (ROS) responsible for dye degradation. Although CN-MgO-76 also exhibits a relatively high degradation efficiency, its rate constant (0.01247 min^–1^) is slightly lower than that of CN-MgO-61. This decrease may be attributed to excess MgO content causing partial surface coverage of the active GCN sites or increased recombination centers, which can limit charge transfer efficiency. Overall, the kinetic analysis clearly demonstrates that CN-MgO nanocomposites possess significantly improved photocatalytic performance compared to the individual MgO and GCN materials, with the CN-MgO-61 composition providing the optimal balance between MgO loading and effective interaction with the GCN matrix.^70–74^ A comparison of CN-MgO-61 with other similar reported work of MgO/GCN composites has been discussed in Table 5. For example, Dahiya et al.^15^ reported 98% degradation of methylene blue within 120 min using a MgO/GCN photocatalyst synthesized from urea and magnesium acetate under xenon lamp irradiation. Similarly, Murugesan et al.^46^ achieved 91% and 98% degradation of crystal violet and reactive black dyes, respectively, while Jaganathan et al.^47^ reported degradation efficiencies of 98.9% for crystal violet and 97.33% for eosin yellow under sunlight irradiation. In contrast, Ikram et al.^49^ obtained a comparatively lower degradation efficiency of 45% for methylene blue/ciprofloxacin within 200 min. The CN-MgO-61 nanocomposite developed in the present study achieved 89% degradation of SO within 120 min under solar simulator irradiation. Although direct comparison is difficult due to differences in pollutants, irradiation sources, and reaction conditions, the results demonstrate that the photocatalytic activity of the present material is comparable with previously reported MgO/GCN systems. Furthermore, the use of orange-fruit peel as a sustainable bioderived precursor provides an environmentally friendly and cost-effective synthesis route.

**Table 4:** Degradation % of SO Dye for the 519 nm Absorbance Peak and Apparent Rate Constant

Sample	Degradation in (%) after 120 min	Apparent rate constant (k) (min^–1^)
**GCN**	43	0.00347
**MgO**	45	0.0044
CN-MgO-28	79	0.01128
CN-MgO-44	84	0.01343
CN-MgO-61	89	0.01609
CN-MgO-76	83	0.01247

**Table 5:** Comparison of Degradation Efficiency of Dyes with a Previously Reported Work

Sl. no	Precursor used for synthesizing MgO/GCN nanocomposites	Synthesis method	Light source	Pollutant type	Degradation efficiency (%)	refs
**1**	Urea and magnesium acetate	Calcination	200 W xenon bulbs	Methylene Blue	98/120 min	^15^
**2**	Melamine, Magnesium chloride anhydrous, Sulfuric acid	Calcination		Crystal violet and Reactive black	91 and 98/120 min	^42^
**3**	Magnesium Chloride, Melamine, Sulfuric acid	Calcination	Under sunlight	Crystal Violet and Eosin Yellow)	98.9 and 97.33/120 min	^43^
**4**	Magnesium chloride, Graphite powder	chemical coprecipitation	NaBH_4_	Methyl blue ciprofloxacin	45/200 min	^45^
**5**	Magnesium acetate and orange peel	Calcination (One step green synthesis method)	Solar simulator	Safranine O	89/120 min	Present work

**Figure 11:**
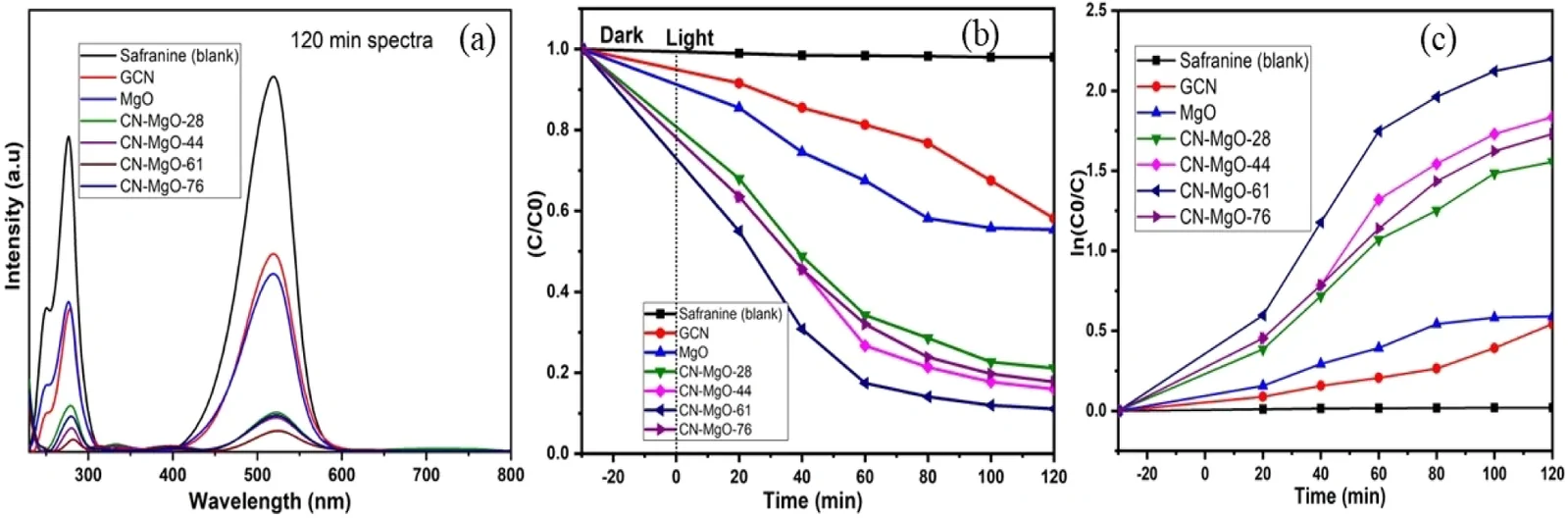
(a) Photocatalytic activity under a solar simulator evaluated by UV–vis absorbance from 200 nm–800 nm for all samples, after 120 min of dye degradation­(b) comparison of photocatalytic degradation activity at 519 nm; and (c) ln­(C_0_/C­(t)) for SO degradation as a function of irradiation time for GCN, MgO, CN-MgO-28, CN-MgO-44, CN-MgO-61, and CN-MgO-76 nanocomposites.

**Figure 12:**
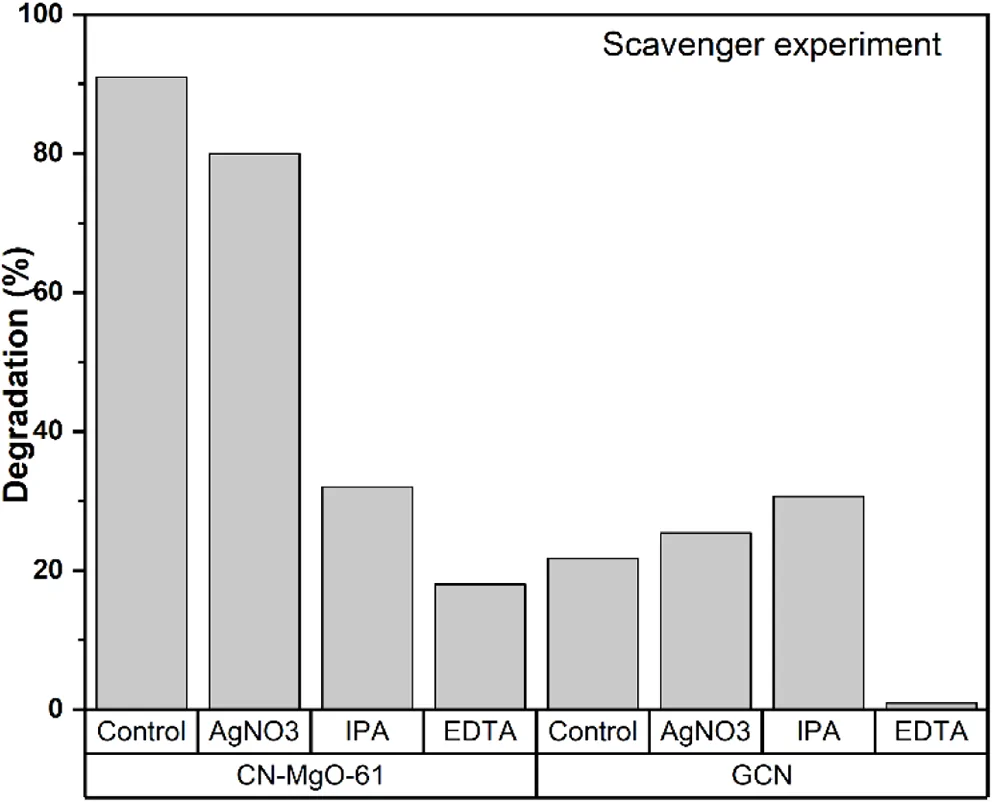
Photocatalytic degradation efficiency of SO in the presence of different radical scavengers (EDTA, AgNO_3_ and isopropanol) and in the presence of GCN or CN-MgO-61 photocatalysts under solar simulator exposure for 120 min.

### Scavenger Experiments and Photocatalytic Mechanism

Radical scavenger experiments were conducted to identify the dominant reactive species involved in the photocatalytic degradation of SO, including photogenerated holes, electrons, and hydroxyl radicals. In Figure 12, the photocatalytic degradation efficiencies of CN-MgO-61 in the presence of a hole scavenger (EDTA), electron scavenger (AgNO_3_), and hydroxyl radical scavenger (IPA) are presented.

In the absence of scavengers (control sample), GCN exhibits relatively low photocatalytic activity (∼22%), which is lower than the degradation efficiency observed in the photocatalytic experiments shown in Figure 11. This discrepancy may arise from differences in catalyst dispersion and aggregation during the scavenger experiments. However, the relative trends observed in the presence of different scavengers remain useful for identifying the dominant reactive species involved in the photocatalytic process. In the presence of isopropanol (IPA), which is commonly used as a hydroxyl radical scavenger, a slight improvement in photocatalytic degradation is observed. However, the addition of IPA also increases the measured pH of the reaction medium from 5.1 to 8.5. It is well-known that an increase in pH enhances the dispersion stability of g-C_3_N_4_ due to surface deprotonation and increased electrostatic repulsion between particles, leading to improved suspension homogeneity and a larger accessible active surface area.^17,18,27,42,44,57,69^ Therefore, the observed increase in photocatalytic activity in the presence of IPA cannot be attributed solely to hydroxyl-radical scavenging. This suggests that hydroxyl radicals are unlikely to be the dominant reactive species in SO degradation with pure GCN under the present conditions. When AgNO_3_ is introduced as an electron scavenger, the photocatalytic degradation increases slightly to ∼25%, while the initial pH decreases to 4.5. Under mildly acidic conditions, surface nitrogen sites of GCN become protonated, promoting particle aggregation and reducing the number of accessible active sites. The slight increase in activity in the presence of AgNO_3_ therefore indicates that electron scavenging suppresses electron–hole recombination, thereby prolonging the lifetime of photogenerated holes responsible for dye oxidation. This interpretation is further supported by the EDTA mediated holescavenger experiments, in which degradation becomes almost negligible and the pH decreases further to ∼3.1. At such low pH values, strong protonation of surface nitrogen functionalities leads to severe agglomeration of GCN particles, significantly limiting the available reactive surface area. Nevertheless, the almost negligible photocatalytic efficiency in the presence of EDTA cannot be attributed to pH alone. Overall, the scavenger results suggest that hole-mediated oxidation plays a significant role in the photocatalytic degradation mechanism of SO over GCN, although the photocatalytic performance is strongly influenced by pH-dependent dispersion effects. Importantly, the pH variations induced by the scavengers may influence the dispersion state and aggregation behavior of GCN, thereby affecting the measured photocatalytic activity. For example, the pH values recorded for GCN, MgO, and CN-MgO-61 in the dye solution before commencing photocatalytic studies were 5.1, 10.6, and 6.7, indicating an acidic environment that could induce GCN aggregation. The pH becomes basic for the MgO nanoparticles. For the nanocomposite the pH becomes almost neutral. This suggests that the GCN is less aggregated, exposing a higher catalytic surface. Therefore, the scavenger experiments alone cannot be considered direct evidence for the charge-transfer pathway or the exact contribution of each reactive species. Rather, the scavenger results should be interpreted together with the structural, spectroscopic, and photocatalytic characterization data. While pH-dependent dispersion effects may partially contribute to the activity variations observed for GCN, they cannot fully account for the substantial enhancement in photocatalytic performance observed after MgO incorporation.

In the absence of any scavenger, CN-MgO-61 exhibits a photocatalytic degradation efficiency of 91%. The pH values obtained in the control and in the presence of reagents for CN-MgO-61were similar to GCN. In the presence of the electron scavenger AgNO_3_ with pH of 4.6, the photocatalytic activity remains relatively high at ∼80%, indicating that electrons play only a minor role in the degradation process, also suggesting that the presence of MgO likely reduces the agglomeration in GCN. The addition of AgNO_3_ scavenges photogenerated electrons, suppressing the formation of superoxide radicals and the subsequent peroxide-mediated pathway to hydroxyl radicals. The relatively small decrease in degradation efficiency indicates that electron-driven ROS pathways play a minor role in the dye degradation process. In contrast, when isopropyl alcohol is introduced as a hydroxyl radical scavenger with a pH of 8.3, the photocatalytic degradation drops significantly to 32%, demonstrating that hydroxyl radicals (•OH) play an important role in the degradation process. Finally, the addition of the hole scavenger EDTA with a pH of 3.5 leads to the lowest photocatalytic activity, indicating that photogenerated holes (h^+^) are the dominant reactive species responsible for SO degradation in the presence of the CN-MgO-61 nanocomposite.

1 $$H_{2}{O+h}^{+}\to \cdot {\mathrm{OH}+H}^{+}$$

2 $${\mathrm{OH}}^{-}{+h}^{+}\to \cdot \mathrm{OH}$$

Holes promote the formation of hydroxyl radicals through the oxidation of water molecules or hydroxyl ions (eqs 1 and 2). In addition, holes can directly oxidize the dye molecules by extracting electrons from the π orbitals of the conjugated system of the phenazinium chromophore that contains a large number of delocalized π electrons, whereupon weakening the molecular bonds.

3 •OH+Dye→intermediates

These oxidized dye molecules are then further attacked by hydroxyl radicals, producing intermediate degradation products (eq 3). Therefore, the primary degradation pathway is hole-driven oxidation, followed by hydroxyl radical attack. Superoxide radicals play a minor role in the overall degradation mechanism and contribute to some hydroxyl radical generation via superoxide pathways, which explains the slight decrease in the photocatalytic efficiency in the presence of electron scavengers.

Increasing the MgO amount, also increases the transparency of the sample allowing for MgO nanoparticles to absorb light especially in the lower layers of the nanocomposite, which is visible through the change of the nanopowder colors from black to gray with increasing MgO content.

The enhanced photocatalytic performance of the CN-MgO nanocomposites is attributed to efficient interfacial charge separation and transfer between GCN and MgO under visible-light irradiation. The strong electronic interaction between the two components is supported by the observed XPS (Figure 6) binding-energy shifts, FTIR (Figure 5) spectral variations, XRD (Figure 2) peak shifts, suppressed photoluminescence intensity, and enhanced photocatalytic activity. Furthermore, the CN-MgO system contains a significant concentration of defect states, including nitrogen vacancies- in GCN and F/F^+^ center-related states in MgO, which introduce additional electronic levels within the band gap that actively participate in charge trapping, migration, and transfer processes (Figure 13). Consequently, the charge-transfer behavior cannot be adequately described using an ideal heterojunction model based solely on the band structures of defect-free materials. Therefore, although literature-reported band positions provide a useful qualitative framework, they are insufficient for establishing the exact charge-transfer pathway in the absence of experimental band-edge determination, such as Mott–Schottky analysis. The enhanced photocatalytic activity and suppressed photoluminescence together indicate efficient defect-mediated interfacial charge separation in the CN-MgO nanocomposites.^70–74^ Similar observations have been reported in recent visible-light-driven heterostructured photocatalysts, where enhanced photocatalytic performance has been attributed to defect-assisted interfacial charge separation and reactive oxygen species generation rather than to a strictly defined heterojunction mechanism. In particular, Rath et al.^31^ demonstrated that spectroscopic evidence, reactive species trapping experiments, and ROS detection analyses collectively support a defect-mediated charge-transfer pathway in complex heterostructured photocatalysts. However, in the absence of direct band-edge measurements, the exact heterojunction classification cannot be conclusively established. Therefore, the photocatalytic mechanism is discussed as a qualitative defect-mediated charge-transfer process rather than being assigned to a definitive Type-I, Type-II, Z-scheme, or S-scheme heterojunction.

**Figure 13:**
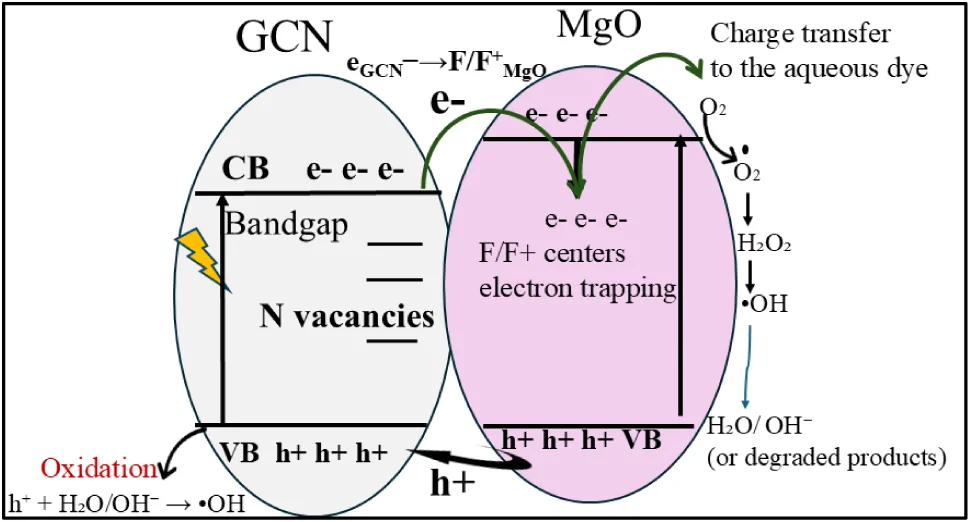
Schematic of defect-mediated charge-separation mechanism inferred from photoluminescence study, photocatalytic properties studies, and scavenger analyses.

## Conclusion

The present study reports the successful green synthesis of highly efficient visible-light-driven CN-MgO nanocomposite photocatalysts via a one-step method using orange peel biowaste. Cytotoxicity studies confirmed that the synthesized nanoparticles and nanocomposites exhibit negligible toxicity toward living cells, which is attributed to the effective embedment of MgO nanoparticles within the GCN matrix. This enhances their suitability for environmental applications.

A clear synergistic effect between GCN and MgO nanoparticles was observed in the photodegradation of SO. Photoluminescence analysis further demonstrated that the CN-MgO nanocomposites significantly improve the separation of photogenerated charge carriers, leading to enhanced photocatalytic performance under visible-light irradiation. The optimized CN-MgO-61 nanocomposite achieved approximately 89% degradation of SO within 120 min under visible light. Since the photocatalytic degradation mechanism is predominantly hole-mediated, the pH of the reaction medium plays an important role in determining the overall photocatalytic efficiency. While hole generation itself is not suppressed at near-neutral pH (∼6), strongly acidic conditions (pH ≤ 4) tend to modify the surface charge, reduce dispersion stability, and limit the availability of hydroxide ions for radical formation, leading to increased recombination losses and reduced photocatalytic activity. Therefore, pH adjustment of the aqueous dye medium is essential for achieving optimal photocatalytic performance with CN-MgO nanocomposites, due to the presence of GCN.

## Supplementary Material



## Notes

Raman peak deconvolution and residual analyses; normalized and deconvoluted Raman spectra; PL peak fitting validation; photocatalytic activity evaluation using UV–Vis absorption spectra; and additional spectral analyses supporting the structural, optical, and photocatalytic properties of CN-MgO samples (PDF)
