# Supplementary material for: Mechanistic Investigation of Visible-Light-Driven Photocatalysis in Bio-Synthesized Graphitic Carbon Nitride/MgO Nanocomposites Toward Safranine Orange Dye
Source: ACS Omega. 2026 Aug 13;11(33):49651–68. doi: 10.1021/acsomega.6c03815 (PMC13520051; doi:10.1021/acsomega.6c03815)
Supplement: Supplementary file 1 [file ao6c03815_si_001.pdf]

Mechanistic Investigation of Visible-Light Driven Photocatalysis in Bio-Synthesized Graphitic Carbon Nitride/MgO Nanocomposites towards Safranine Orange dye

Priyanka Panchal[1], Protima Rauwel[2], Laura Coconubo-Guio[1], Glemarie Hermosa[1], Shubham Gautam[3], Satya Pal Nehra[4], Elias Estephan [5,6], Erwan Rauwel[1*]*

1. Institute of Veterinary Medicine and Animal Sciences, Estonian University of Life Sciences, 51006 Tartu, Estonia
2. Department of Aeronautical Engineering, Estonian Aviation Academy, 61707 Tartu, Estonia
3. Materials Research Center, Malaviya National Institute of Technology, Jaipur 302017, India
4. Center of Excellence for Energy and Environmental Studies, Deenbandhu Chhotu Ram University of Science and Technology, Sonipat 131039, India
5. Laboratoire Bioingénierie et Nanoscience (LBN), University of Montpellier, Montpellier, France
6. Faculty of Engineering, Sagesse University, Furn El Chebbak, Baabda, Lebanon

E-mail: protima.rauwel@eava.ee, erwan.rauwel@emu.ee

Supporting Information

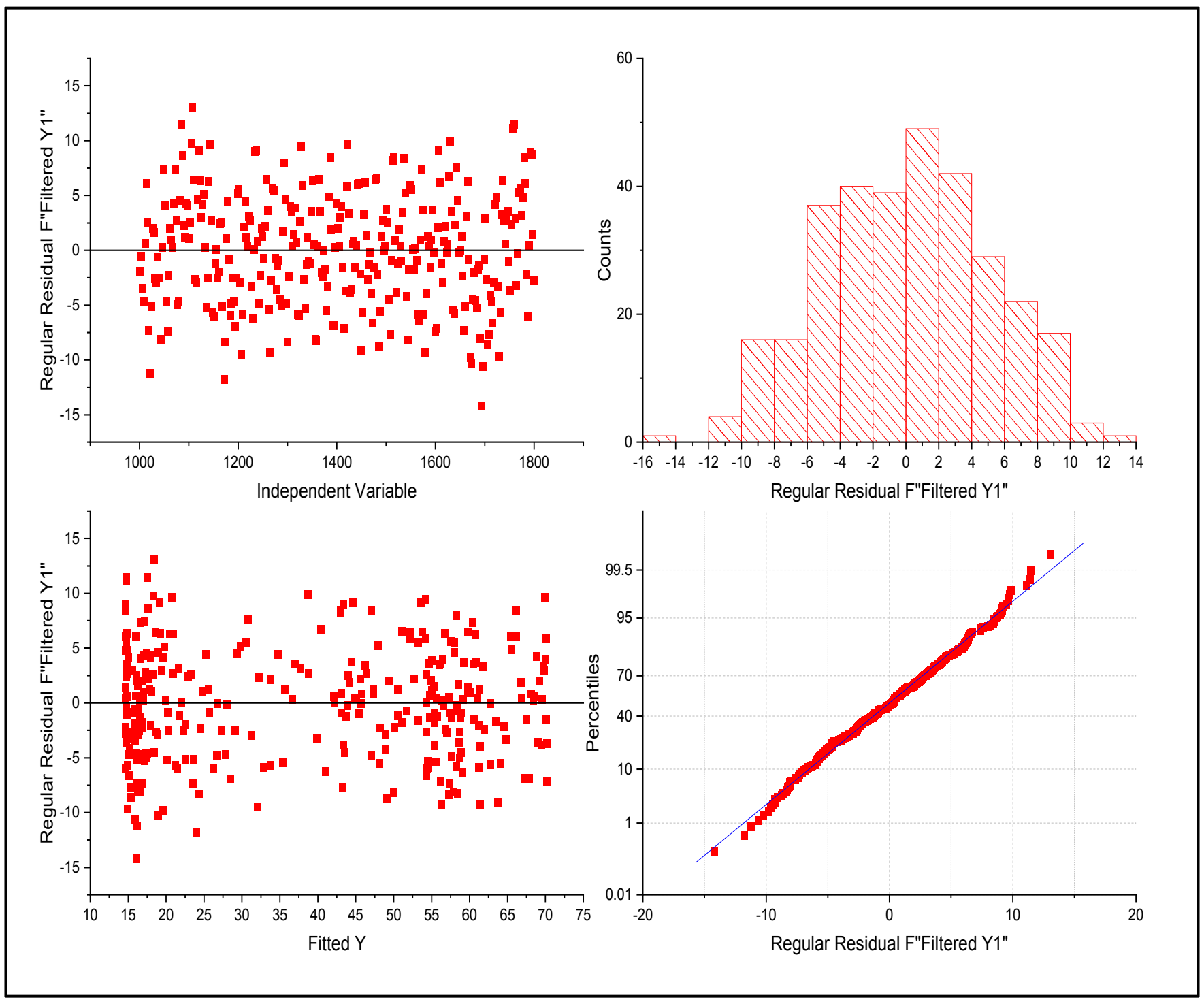

Figure S1. Representative residual analysis of the Raman peak deconvolution. Residuals plotted as a function of Raman shift (top left) and fitted intensity (bottom left), together with the residual distribution histogram (top right) and normal probability plot (bottom right). The random distribution of residuals about zero and their approximately normal.

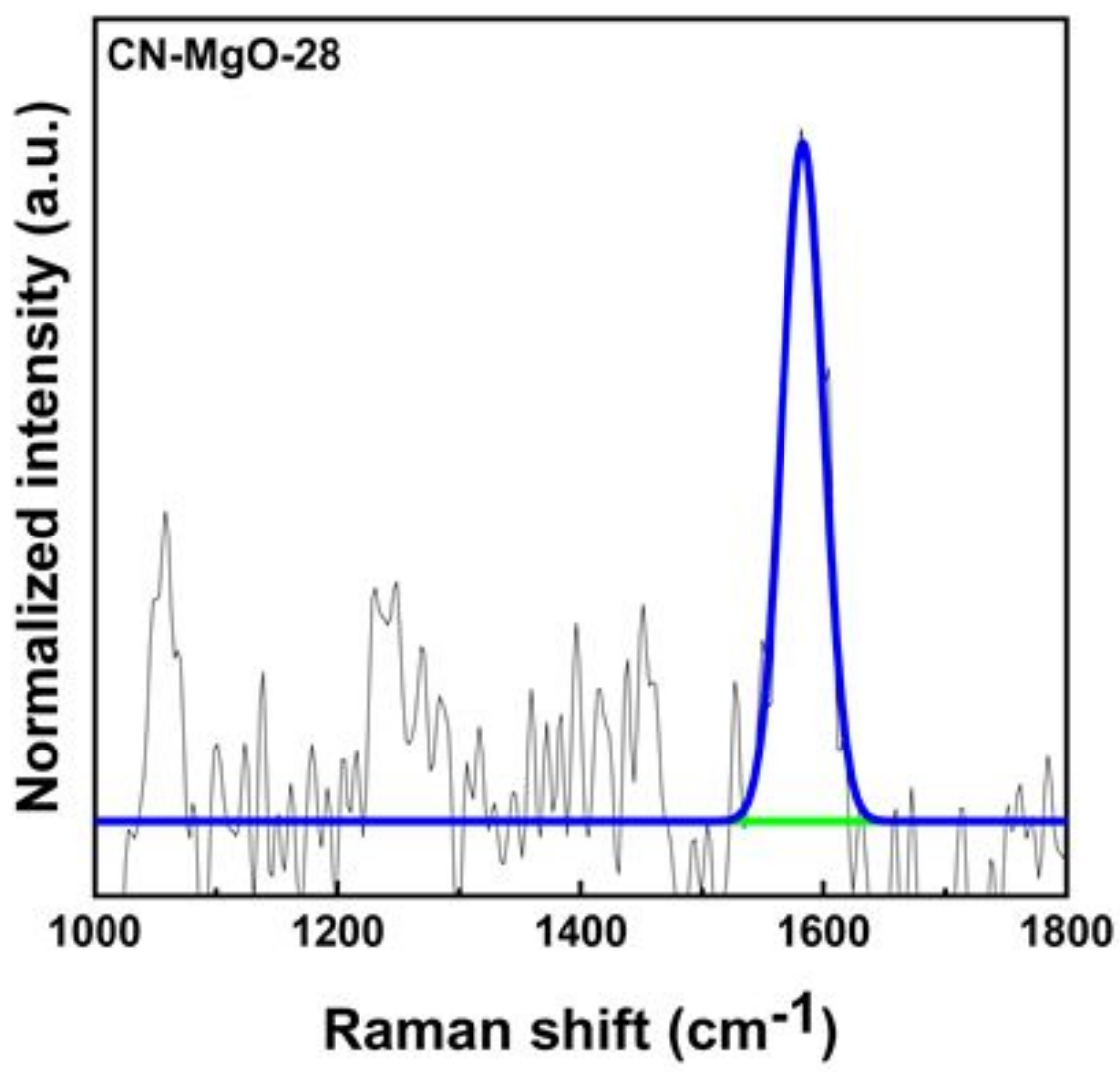

Figure S2. Normalized and deconvoluted Raman spectra of CN-MgO-28

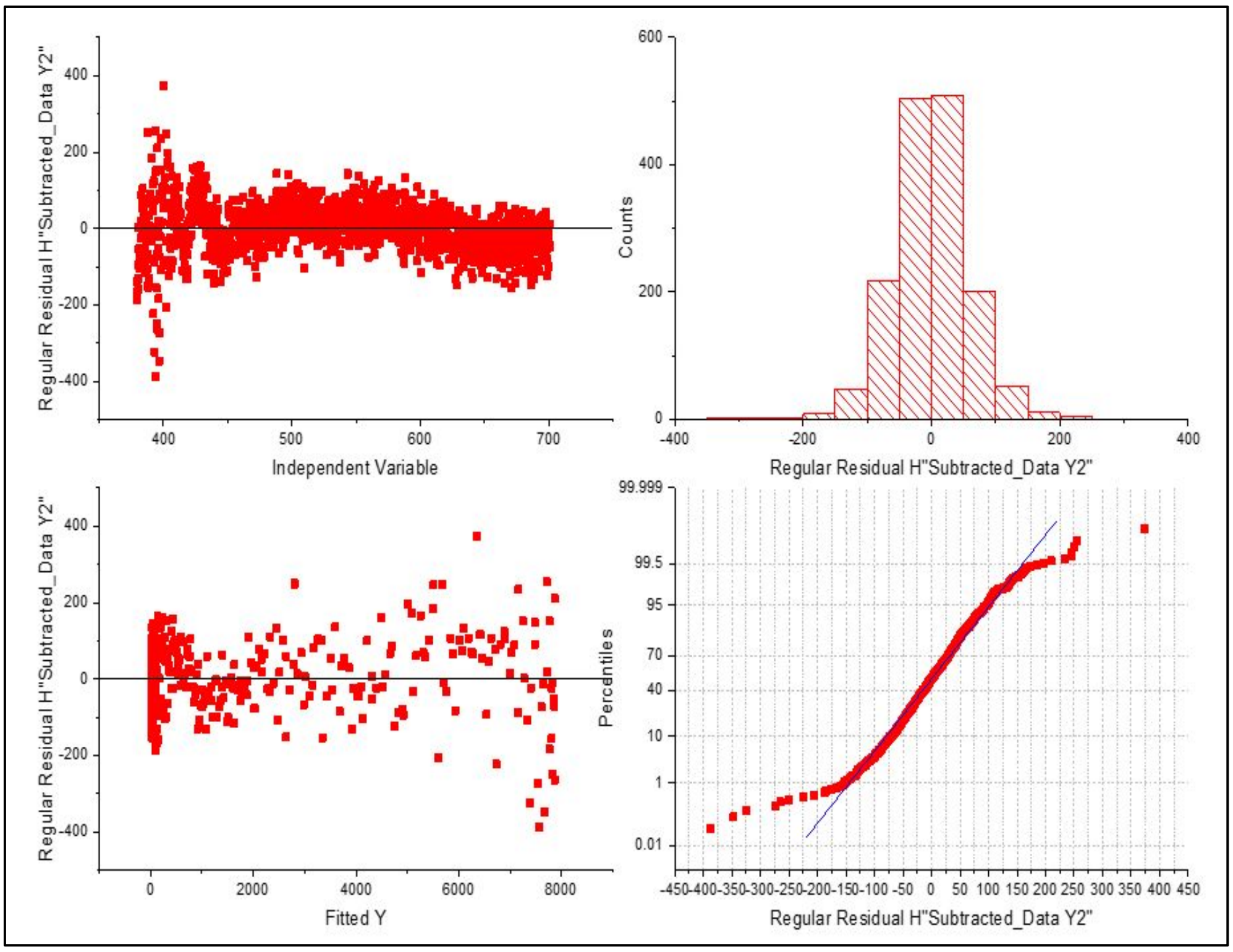

Figure S3. Residual analysis of a representative PL spectrum following peak deconvolution. The residuals are plotted as a function of wavelength (top left) and fitted intensity (bottom left), together with the residual distribution histogram (top right) and normal probability plot (bottom right). The residuals are centered about zero and show no significant systematic deviations, indicating that the fitted model adequately describes the experimental photoluminescence spectrum. The same fitting procedure and constraints were applied to all PL spectra analyzed in this work.

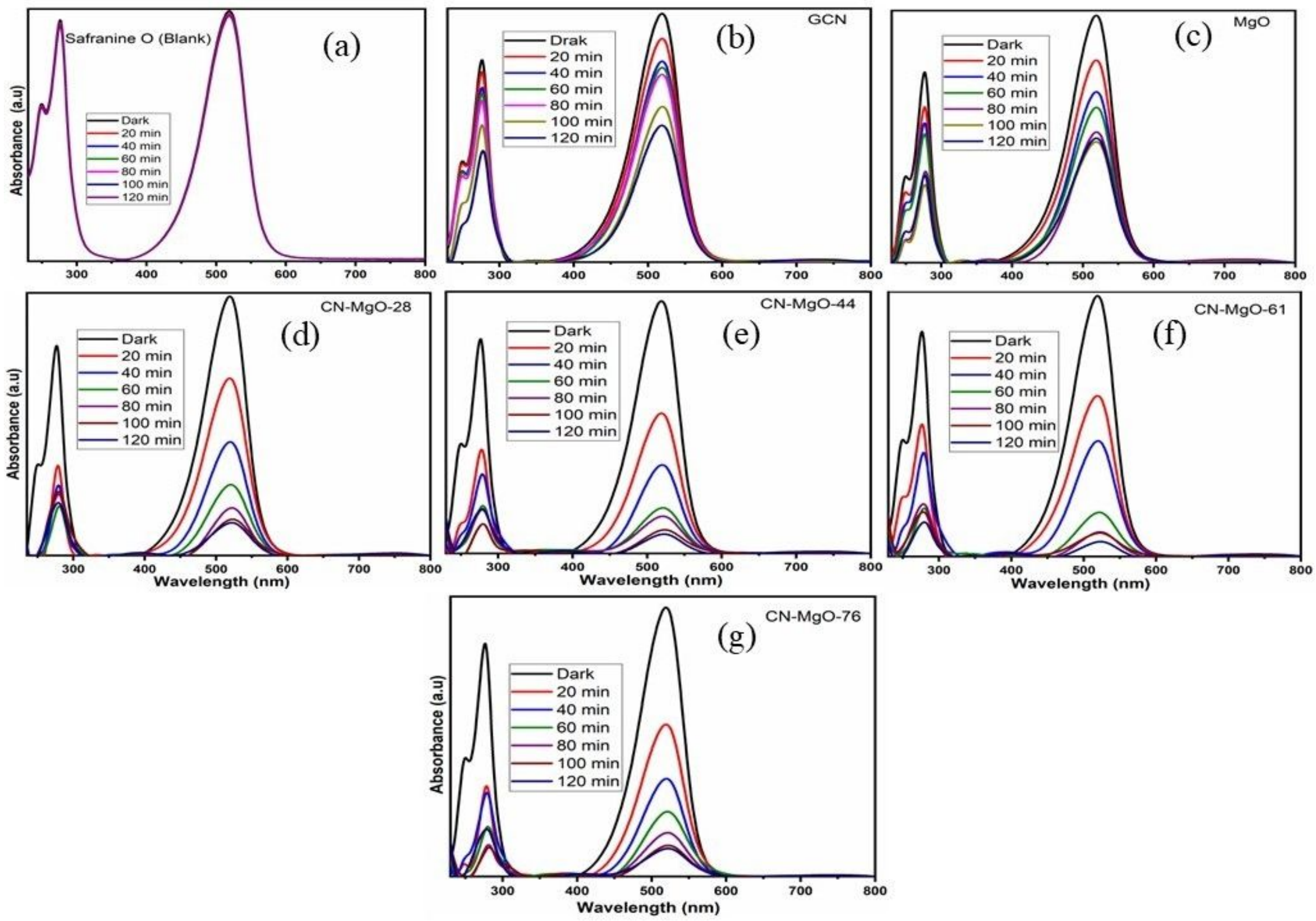

Figure S4. Photocatalytic activity under a solar simulator evaluated by UV-Vis absorbance from 200 nm-800 nm for(a) safranine O, (b) GCN, (c) MgO, (d) CN-MgO-28, (e) CN-MgO-44, (f) CN-MgO-61, (g) CN-MgO-76.
